# Green Synthesis and Functional Design of Polypyrrole-Based Nanomedicines for Cancer Theranostics: A Critical Review and Sustainability-Guided Perspective

**DOI:** 10.3390/polym18162030

**Published:** 2026-08-21

**Authors:** Jiaqiao Zhong, Yuanzhe Li

**Affiliations:** 1 West China Hospital, Sichuan University, Chengdu 610065, China; zhongjiaqiao0614@163.com; 2 Carbon Neutrality Institute, China University of Mining and Technology, Xuzhou 221116, China; 3 NUS College of Design and Engineering, National University of Singapore, Singapore 117575, Singapore

**Keywords:** polypyrrole nanomedicine, green synthesis, cancer theranostics, photothermal–photodynamic synergy, metal–polyphenol coordination

## Abstract

Nanomedicine has advanced cancer theranostics via targeted delivery and phototherapy, yet many high-performance systems rely on inorganic or metal-intensive materials synthesized through energy-demanding routes, raising concerns about biocompatibility, environmental accumulation, and sustainability. This review re-evaluates polypyrrole (PPy)-based nanomedicines from a green chemistry perspective, shifting focus from performance-centric optimization to sustainability-guided design. PPy, an organic conductive polymer with near-infrared photothermal activity and structural tunability, offers a promising platform. However, pristine PPy suffers from limited functionality, poor biodegradability, and insufficient reactive oxygen species (ROS) generation. Reported FeCl_3_-, CuCl_2_-, and Fe^2+^/H_2_O_2_-mediated routes are compared to examine formulation-specific relationships among synthesis conditions, polymer characteristics, redox behavior, ROS-related function, and process burdens. Because the underlying studies differ in composition, processing, purification, and assay conditions, these comparisons are used to identify evidence-supported trade-offs and data gaps rather than to establish a universal causal hierarchy. Green strategies are critically assessed, including one-step carboxylated copolymerization for backbone degradability and metal–polyphenol networks for catalytic ROS amplification. To organize the heterogeneous evidence, this review introduces a PPy-specific dual-axis evidence map that considers process-related sustainability alongside biofunctional performance. This qualitative tool is intended to identify trade-offs and evidence gaps rather than provide a validated sustainability score.

## 1. Introduction

Cancer remains a major global health burden, with approximately 20 million new cases and 10 million cancer-related deaths estimated worldwide in 2022 [1]. Nanomedicine offers opportunities to improve tumor localization [2], controlled delivery [3], and externally triggered therapy [4]. Among these approaches, photothermal therapy (PTT) and photodynamic therapy (PDT) have received particular attention because they can provide spatially controlled treatment and can be combined to address incomplete tumor ablation and limitations inherent to either modality alone [5,6,7]. However, the development of cancer nanomedicines has remained predominantly performance-oriented, with therapeutic efficacy often discussed more extensively than material sourcing, synthetic burden, biological persistence, and end-of-life considerations [8,9,10].

This imbalance is relevant because high therapeutic performance does not necessarily imply sustainable material design [11]. Many widely studied nanotherapeutic systems contain noble metals, semiconductor components, transition-metal compounds, or rare-earth-based elements whose preparation may require energy-intensive processing, hazardous reagents, organic solvents, and multiple purification steps [10,12,13]. Their non-degradable cores may also undergo prolonged biological retention, although fate and toxicity remain strongly dependent on formulation, dose, and exposure conditions [14]. Organic photothermal platforms, including small-molecule dyes, polydopamine, semiconducting polymers, and conductive polymers, can reduce reliance on persistent inorganic cores [15]. However, they are not automatically sustainable: solvent use, multistep formulation, colloidal stability, degradation products, and clearance must still be evaluated. Sustainability comparisons should therefore be formulation- and process-specific rather than based solely on whether a material is classified as organic or inorganic [16,17,18,19].

These considerations support a shift from performance-driven optimization toward design approaches that integrate function, safety, manufacturability, and lifecycle impacts [11]. Green chemistry metrics, lifecycle assessment, Safe-by-Design, and Safe and Sustainable by Design provide established principles for evaluating material and process sustainability [10,16,20,21]. However, the systematic application of these approaches to PPy-based nanomedicines remains limited. Existing studies use diverse synthesis, purification, functionalization, and biological testing protocols, while reporting of material inputs, purification burden, degradation, metal release, and long-term biological fate is frequently insufficient for direct cross-study sustainability comparison [22,23,24,25,26,27]. The principal gap is therefore not the absence of general sustainability frameworks, but the lack of a PPy-specific structure for organizing the available evidence while clearly distinguishing measured results, indirect evidence, and unresolved data gaps.

In this context, polypyrrole (PPy) offers a potentially greener starting point than many metal-intensive photothermal nanomaterials, as its organic conjugated backbone supports near-infrared (NIR) photothermal conversion, and can be formed via relatively mild aqueous oxidative polymerization [24,28,29]. Its structural tunability also enables the integration of photothermal, catalytic, drug-delivery, and imaging functions within a single platform [23,30,31]. Nevertheless, sustainability is not an intrinsic property of PPy itself [10,21]. Pristine PPy has limitated reactive functionality [32], weak oxygen species (ROS) generation [33], uncertain long-term degradation and clearance [34,35], and may require additional coatings, dopants, metals, or purification steps that partly offset the advantages of the initial synthesis [25,30]. This review, therefore, examines how the synthesis route and functional design are associated with both process-related sustainability and biofunctional performance. To organize these heterogeneous data, we introduce a PPy-specific dual-axis evidence map. The framework is intended as a qualitative evidence-organizing tool for identifying trade-offs and reporting gaps, rather than as a validated sustainability score or a replacement for established green chemistry, lifecycle, or SSbD assessments [10,21].

This article adopts a critical narrative review approach rather than a systematic review or meta-analysis [36]. Relevant publications were identified through targeted searches of Web of Science, Scopus, PubMed, and Google Scholar, supplemented by backward and forward citation tracking of key studies. The literature considered primarily covered publications available up to June 2026, while earlier seminal studies were retained where they established fundamental concepts concerning PPy synthesis, photothermal and redox behavior, degradation, metal-associated effects, biological safety, or translational feasibility. Priority was given to studies providing experimentally supported mechanistic or quantitative evidence; high-quality reviews were used mainly to establish context and identify relevant primary research. Studies lacking sufficient methodological detail, direct relevance to biomedical PPy systems, or experimental support for sustainability-related claims were not emphasized. Given the heterogeneity of formulations, experimental conditions, biological models, and reporting practices, this review does not claim exhaustive coverage. However, it provides a structured critical synthesis of recurring evidence patterns, unresolved trade-offs, and key reporting needs.

## 2. Green Chemistry Principles Applied to Nanomedicine Synthesis

Polypyrrole (PPy) is examined here not simply for its photothermal efficiency, but as a model organic nanomaterial through which nanomedicine design can be reinterpreted using green chemistry and safe-and-sustainable-by-design concepts. This perspective shifts attention from isolated efficacy metrics toward the reported relationships—and, where direct comparative evidence remains unavailable, plausible rather than proven mechanistic connections—among synthesis pathway, nanostructure, biological function, manufacturing burden, and post-use fate [37,38].

Compared with many hard, poorly degradable nanotherapeutics, metal-free PPy formulations lack a persistent metallic core. Nevertheless, organic composition alone should not be treated as evidence of biodegradability or rapid biological clearance. The liver and spleen efficiently sequester hard nanomaterials, and gold nanoparticles may remain within hepatic cell populations for prolonged periods. However, such retention does not necessarily result in overt toxicity in all formulations [39,40]. For PPy, long-term degradation and elimination remain formulation dependent. Notably, renal clearance has been demonstrated for deliberately engineered ultrasmall PPy–PEG nanoparticles of approximately 2 nm in diameter, rather than for PPy as a material class [35].

PPy exhibits broad near-infrared (NIR) absorption arising from its conjugated and doped electronic structure, enabling photothermal conversion without relying on plasmonic metals. Representative PPy formulations have been reported to achieve photothermal conversion efficiencies of 33.35% and 44.7% at 808 nm [24,35]. Uniform PPy nanoparticles have also shown photothermal performance and photostability that are competitive with those of Au nanorods under the specific experimental conditions tested [24]. However, comparisons across studies should be interpreted with caution because reported conversion efficiency depends on particle concentration, optical extinction, laser power density, irradiation geometry, heat-loss assumptions, and the calculation protocol employed.

PPy is commonly prepared by chemical oxidative polymerization, allowing particle formation, colloidal stabilization, and functionalization to be combined during material synthesis. One-pot aqueous PVP-assisted polymerization and recent in situ nanocaging or crosslinking strategies illustrate how colloidal stability and therapeutic functionality can be introduced concurrently with PPy formation [41,42]. Such process integration is consistent with step economy and may reduce the use of auxiliary reagents and post-synthetic operations. Nevertheless, a one-pot process should not automatically be classified as environmentally preferable unless its conversion, isolated yield, reagent demand, purification burden, and waste generation have been quantified.

Pristine or minimally functionalized PPy is therefore not inherently sustainable. It provides relatively few readily accessible chemical handles; its degradation and long-term biological fate remain insufficiently characterized, and strong reactive oxygen species (ROS)-generating activity generally requires incorporating a photosensitizer or another redox-active component. For example, coupling PPy with methylene blue enabled combined photothermal and photodynamic activity through photosensitizer-mediated ROS generation [31]. The central challenge is consequently to redesign PPy synthesis and architecture so that optical performance, therapeutic function, process efficiency, and post-treatment fate are considered together.

### 2.1. Green Chemistry Criteria for Nanomedicine Evaluation

To enable a more rigorous assessment of PPy-based nanomedicines, general green chemistry principles must be translated into criteria that are directly applicable to nanoscale biomedical systems. Although the classical twelve principles of green chemistry provide the conceptual foundation, not all of them can be readily operationalized using the information typically reported in nanomedicine studies. Accordingly, the present analysis prioritizes criteria that directly influence synthesis, material performance, lifecycle behavior, safety, and translational feasibility, consistent with emerging safe-and-sustainable-by-design frameworks [37,38].

One immediate consideration is the use of mild reaction conditions. In practical terms, reactions conducted at or near ambient pressure and at relatively low temperatures may serve as preliminary indicators of reduced process severity. Representative PPy nanoparticles have been synthesized through one-pot aqueous dispersion polymerization at 4 °C, while other recent PPy architectures have been produced through aqueous FeCl_3_-mediated oxidative polymerization [41,42]. These approaches avoid the high temperatures and pressures associated with many solvothermal syntheses of inorganic nanoparticles. Nevertheless, reaction temperature alone is an incomplete measure of energy efficiency. Mixing, cooling, purification, repeated centrifugation or dialysis, solvent removal, drying, sterilization, and scale-dependent process control may contribute substantially to the overall energy and material burden [43,44,45].

Oxidant selection is another potentially important contributor to polymerization kinetics, polymer composition, doping state, particle morphology, and subsequent material behavior. Experimental studies demonstrate that oxidant concentration, temperature, dopant identity, and reaction conditions can alter pyrrole conversion, PPy morphology, conductivity, stability, and optical properties [46,47]. However, downstream photothermal performance, ROS generation, biological response, and process burden also depend on monomer composition, templates, stabilizers, particle size, surface chemistry, purification conditions, residual-metal content, and measurement protocols. These outcomes should therefore not be inferred solely from oxidant identity.

FeCl_3_- and CuCl_2_-associated routes may differ in reagent hazards, residual metal burden, redox behavior, and metal management requirements. Nevertheless, their overall environmental or biological risks cannot be ranked without formulation-specific measurements of oxidant utilization, residual-metal content, metal speciation, ion release, purification inventories, exposure, and biological disposition [37,38]. Oxidant identity should consequently be treated as one design variable within a multivariable synthesis–structure–function relationship, rather than as an independent predictor of sustainability.

Solvent selection represents a further critical dimension. Aqueous-phase synthesis, widely used for PPy nanoparticle preparation, avoids use of hazardous organic solvents such as dimethylformamide or toluene [41,42,44]. However, water should not be automatically classified as a green solvent without considering its source, required purity, total volume, contamination, treatment requirements, and the energy required for concentration or removal [44,45]. Solvent choice should therefore be assessed at the process level rather than solely according to solvent identity.

Alongside solvent choice, minimizing post-synthetic modification is an important design objective. One-step or in situ functionalization can reduce the number of reagents, reaction vessels, solvent exchanges, and purification operations required to obtain a functional nanomedicine [41,42]. These benefits should ideally be demonstrated using quantitative indicators such as process mass intensity, E-factor, solvent intensity, isolated yield, number of washing cycles, and energy demand, rather than inferred from the number of reaction steps alone [43].

Finally, degradability and multifunctionality are important but distinct criteria for evaluating the sustainability of nanomedicine. A formulation that remains sufficiently stable during storage and systemic administration but undergoes controlled disassembly, degradation, or elimination after fulfilling its therapeutic function may offer advantages for both efficacy and long-term safety. However, degradability must be demonstrated through direct identification of degradation kinetics, products, residual fragments, and biological clearance rather than inferred from organic composition or stimulus responsiveness alone [35].

Similarly, integrating therapeutic and diagnostic functions into a single material system may reduce the number of separately manufactured components and simplify administration. Conversely, each additional photosensitizer, metal ion, targeting ligand, imaging agent, or coating may increase reagent demand, purification complexity, analytical requirements, and uncertainty regarding biological fate. Multifunctionality should therefore be evaluated in terms of useful function delivered per unit of material and process burden, rather than by counting the number of functions incorporated [37,38,43].

Taken together, these criteria establish a synthesis-informed framework for evaluating PPy nanomedicines. The framework shifts attention from isolated performance indicators toward the combined assessment of reaction conditions, reagent and solvent selection, process mass efficiency, purification demand, residual species, therapeutic function, degradability, and long-term fate.

### 2.2. Conceptual Positioning of the PPy-Specific Dual-Axis Framework Within Established Sustainability Assessment Approaches

Green chemistry provides the process-level foundation for reducing hazardous substances, energy consumption, auxiliary materials, and waste. Its twelve principles are complemented by mass-based metrics, including atom economy, the E-factor, and process mass intensity, which quantify different aspects of synthetic efficiency as summarized in Table 1 [21,48]. However, these metrics do not independently capture exposure, biological fate, or environmental burdens across the complete material life cycle. Lifecycle approaches, therefore, extend the assessment boundary from raw-material acquisition and manufacturing to use and end-of-life [49,50]. In nanotechnology, greener nanoscience and sustainable nanotechnology further emphasize integrating hazard reduction and lifecycle considerations during material design rather than evaluating them only after synthesis [51,52].

Safe-by-Design and Safe and Sustainable by Design (SSbD) frameworks broaden this perspective by considering material functionality together with human and environmental safety, process-related risks, circularity, and lifecycle impacts. The revised European Commission SSbD framework incorporates safety assessment, supply-chain and process risks, environmental lifecycle assessment, socioeconomic considerations, and the explicit treatment of trade-offs and uncertainty [53]. The OECD Safe(r) and Sustainable Innovation Approach further connects early-stage material design with regulatory preparedness and information exchange across the innovation chain [54]. Although these frameworks provide comprehensive assessment structures, their implementation requires hazard, exposure, lifecycle inventory, and end-of-life data that are often incomplete for most emerging PPy nanomedicines.

Existing reviews of PPy-based biomedical materials have primarily focused on synthesis, morphology, photothermal performance, and multifunctional therapeutic applications [55]. In the studies examined here, information on monomer conversion, purification inputs, residual-metal mass balances, degradation-product identity, pharmacokinetics, and clearance is frequently missing or reported under non-comparable conditions. Consequently, a robust LCA or complete SSbD assessment cannot yet be performed across the reviewed PPy systems. The present dual-axis approach, therefore, serves a narrower purpose: it maps directly reported process-related sustainability indicators against biofunctional outcomes while explicitly identifying missing, indirect, or non-comparable evidence. Its originality lies in the PPy-specific organization and interpretation of available evidence, rather than in replacing established sustainability frameworks or generating a validated composite score.

**Table 1:** Comparison of established sustainability assessment approaches with the PPy-specific dual-axis evidence-mapping framework.

Approach	Main Scope	Strength	Current Limitation for PPy Assessment
Green chemistry principles and process metrics	Hazard prevention, reagents, solvents, energy consumption, atom economy, process mass intensity (PMI), and E-factor	Evaluates the material and process efficiency of individual synthetic routes [48]	Quantitative application is limited because complete reagent inputs, product yields, purification inputs, and waste outputs are rarely reported for PPy nanomedicines [16,21]
Lifecycle assessment (LCA)	Raw materials, production, use and end-of-life	Extends environmental assessment beyond the synthesis stage and enables comparison across defined system boundaries [56]	A robust PPy-specific LCA cannot currently be conducted because consistent lifecycle-inventory data are unavailable across the studies reviewed [57]
Safe-by-Design and Safe and Sustainable by Design (SSbD)	Functionality, human and environmental safety, process risks, circularity, and lifecycle sustainability	Integrates safety and sustainability considerations during material and process development [20]	Implementation requires hazard, exposure, process, and lifecycle data that remain incomplete or non-comparable for most PPy nanomedicines [53]
PPy-specific dual-axis evidence-mapping framework	Process-related sustainability indicators and biofunctional performance	Organizes available PPy evidence, identifies trade-offs, and explicitly records missing or indirect evidence [55]	Qualitative and not yet validated as a quantitative scoring framework [35]

### 2.3. A PPy-Specific Dual-Axis Evidence Map

The evidence map is organized into two linked domains, reflecting the need to evaluate process-related sustainability separately from, but in connection with, biological performance, safety, and post-administration fate [58]. The process-related sustainability domain records solvent identity, reaction conditions, oxidant identity and hazard, synthesis step count, purification burden, monomer conversion, and process mass intensity (PMI) or E-factor when these parameters are directly reported. The biofunctional domain records photothermal performance, experimentally identified ROS pathways, evidence of structural degradation or disassembly, metal loading and release, cytocompatibility, biodistribution, and clearance [59,60,61].

The map is descriptive rather than aggregative and does not generate a composite greenness score. Parameters not reported in the original study are designated as “NR” and are not estimated or imputed from analogous formulations. This rule is intended to preserve reporting transparency and prevent the conversion of evidence gaps into apparently quantitative comparisons [59,62]. Differences among FeCl_3_-, CuCl_2_-, and Fe^2+^/H_2_O_2_-associated systems are treated as study-specific associations unless supported by direct comparative experiments conducted under sufficiently controlled conditions. To make the strength and relevance of the available evidence explicit, each claim is classified by the authors as: (i) direct PPy evidence, derived from experiments performed on the PPy formulation under discussion; (ii) indirect PPy evidence, derived from a different PPy formulation or experimental context; (iii) analogous-material evidence, extrapolated cautiously from another material class; or (iv) an author-proposed hypothesis requiring experimental validation.

**Figure 1:**
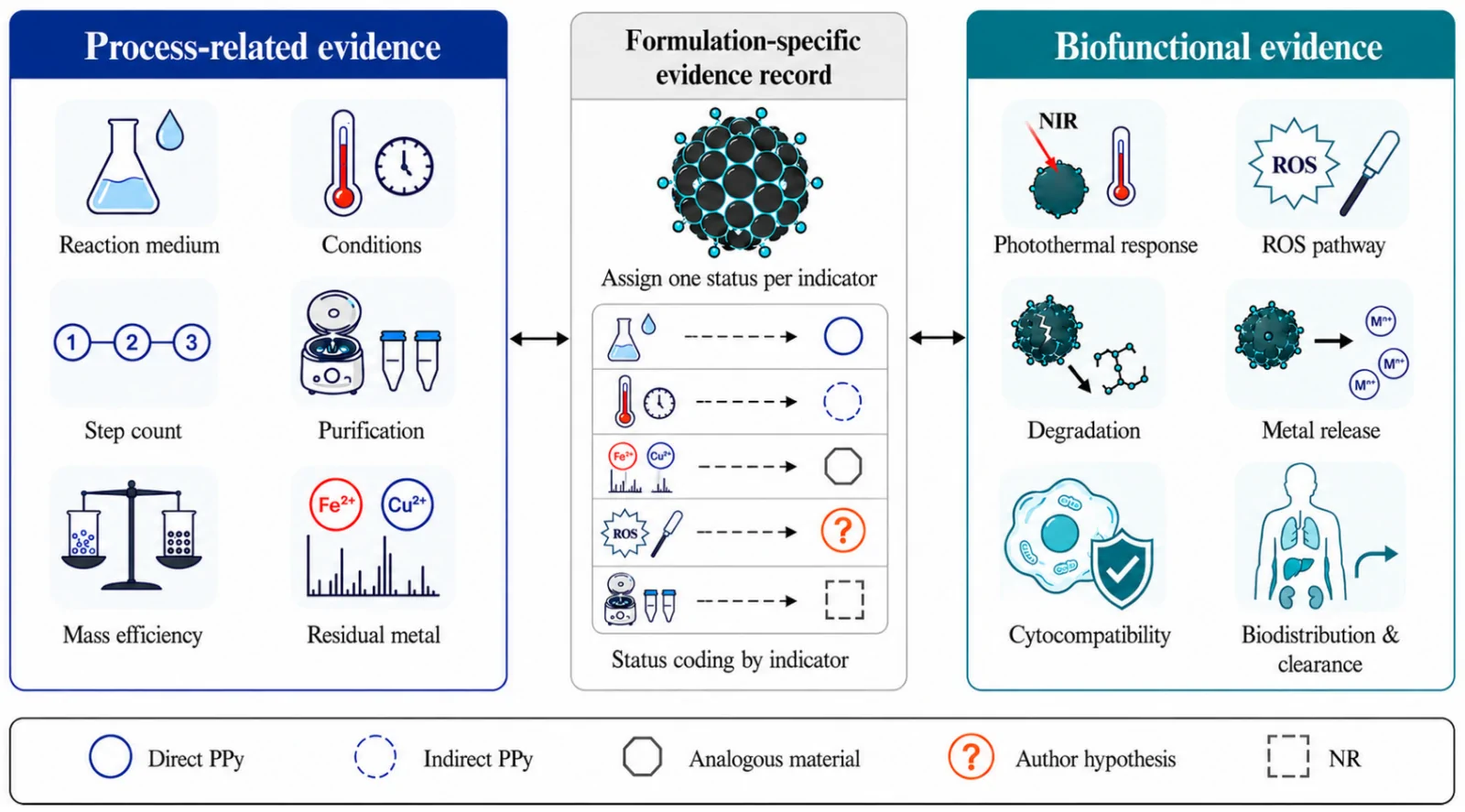
PPy-specific dual-axis evidence map integrating process-related sustainability indicators with biofunctional evidence. The process-related and biofunctional domains define the indicators to be evaluated for each PPy formulation, while the central examples illustrate how one evidence status is assigned to each indicator. These symbols describe evidence provenance and reporting status rather than performance as favorable or unfavorable. The map is intended to support evidence organization and gap identification and does not generate a composite sustainability score or quantitative ranking of PPy formulations or synthetic routes.

As illustrated in Figure 1, the two evidence domains are linked through a formulation-specific record in which each indicator is assigned an evidence-status code. The map is used as a qualitative evidence-organizing tool rather than for the quantitative placement or ranking of individual PPy formulations. Differences among independently reported FeCl_3_-, CuCl_2_-, and Fe^2+^/H_2_O_2_-derived systems are therefore treated as cross-study associations unless supported by controlled within-study comparisons. Detailed route-specific evidence and its associated uncertainties are discussed in Section 4.

### 2.4. Proposed Minimum Reporting Checklist and Quantitative Evaluation Protocol

Qualitative descriptors such as aqueous synthesis, mild temperature, or reduced step count can indicate potential process advantages, but they cannot substitute for atom economy, process mass intensity (PMI), E-factor, or lifecycle assessment. These metrics address different aspects of material efficiency and depend on explicitly defined process boundaries, product composition, and treatment of water, recovered materials, and coproducts [16,21,63,64,65,66,67]. We therefore propose the PPy-specific minimum reporting protocol summarized in Table 2. It adapts established green-metric methodologies, the tiered CHEM21 approach, and minimum-information principles developed for bio–nano research [16,44,59,65,68]. The protocol is an author-proposed reporting framework, not a validated composite sustainability score.

Tier I establishes the batch inventory and reference flow. Studies should report batch scale and the mass of all monomers, oxidants, dopants, metal salts, templates, stabilizers, solvents, water, and washing or dialysis media from reagent charging through purification and drying. The mass of the isolated product should be reported on a defined dry basis, together with drying conditions and, where relevant, water, dopant, and metal contents. Monomer conversion should be determined from a direct mass balance or a validated analytical measurement, rather than inferred solely from the isolated product mass. PPy studies have demonstrated that conversion and isolated yield vary substantially with oxidant concentration, temperature, dopant, and processing route [46,69,70]. Atom economy should therefore be calculated only when the reaction stoichiometry and composition of the desired doped PPy product are sufficiently defined; otherwise, it should be designated as not calculable rather than estimated from an idealized pyrrole repeat unit [21,63,67].

Tier II converts the inventory into auditable process and functional metrics. PMI and E-factor should identify whether water is included, while water and organic-solvent intensities should also be reported separately [16,21,63,64,65,66]. Energy reporting should cover reaction, stirring, sonication, centrifugation, hydrothermal treatment, and drying, distinguishing measured electricity consumption from estimates based on rated equipment power [44,65]. For metal-containing PPy, final metal content and the metal present in supernatants and washing fractions should be quantified; feed ratio alone does not establish incorporation or retention [59,68,71,72]. Photothermal measurements should report wavelength, incident power at the sample, absorbance or extinction, concentration, volume, optical geometry, solvent background correction, and complete heating–cooling data [73,74,75]. ROS measurements should identify the reactive species, the probe, the calibration or reference reaction, interference controls, and the replicate number, because non-specific fluorescent signals cannot be interpreted as a universal quantitative measure of “total ROS” [76].

Tier III extends assessment to degradation, biological safety, and lifecycle boundaries. Degradation claims should combine structural observations with molecular-weight changes, mass loss, organic-carbon balance, degradation-product identification, and metal release, where applicable; morphological collapse alone does not demonstrate polymer mineralization or biological clearance [59,68]. Biological studies should report material characterization in the exposure medium, dose, exposure time, controls, replicate structure, and relevant safety endpoints, with animal experiments additionally following established reporting guidance [68,77]. A screening cradle-to-gate lifecycle assessment should specify its system boundary, electricity mix, allocation assumptions, and upstream reagent production, purification, drying, and waste treatment, in accordance with ISO 14040:2006 (*Environmental Management—Life Cycle Assessment—Principles and Framework*. International Organization for Standardization: Geneva, Switzerland, 2006) and ISO 14044:2006 (*Environmental Management—Life Cycle Assessment—Requirements and Guidelines*. International Organization for Standardization: Geneva, Switzerland, 2006) [78]. For synthesis comparisons, 1 g of dry, application-ready formulation may be used as an initial mass-based reference flow, where equivalent therapeutic function has been demonstrated under harmonized conditions; a performance-normalized result should be reported alongside it. Green-synthesis and biofunctional outcomes should remain separate rather than being combined through subjective weighting.

Table Notes:

$$X_{\mathrm{monomer}}=\left[\frac{n_{0}-n_{\mathrm{residual}}}{n_{0}}\right]\times 100\%;$$

$${\mathrm{PMI}}_{\mathrm{total}}=\frac{\sum m_{\mathrm{inputs}}}{m_{\mathrm{dry}\;\mathrm{product}}};$$

$$E\text{-}\mathrm{factor}=\frac{m_{\mathrm{waste}}}{m_{\mathrm{dry}\;\mathrm{product}}};$$

$$\mathrm{Water}\;\mathrm{intensity}=\frac{m_{\mathrm{water}}}{m_{\mathrm{dry}\;\mathrm{product}}};$$

$$\mathrm{Solvent}\;\mathrm{intensity}=\frac{m_{\mathrm{organic}\;\mathrm{solvent}}}{m_{\mathrm{dry}\;\mathrm{product}}};$$

$$\mathrm{Energy}\;\mathrm{intensity}=\frac{\sum \left(P_{k}t_{k}\right)}{m_{\mathrm{dry}\;\mathrm{product}}};$$

$$\mathrm{Metal}\;\mathrm{retention}=\left(\frac{m_{\mathrm{metal},\mathrm{product}}}{m_{\mathrm{metal},\mathrm{charged}}}\right)\times 100\%;$$

$$\mathrm{Metal}\;\mathrm{balance}\;\mathrm{closure}=\left[\frac{m_{\mathrm{metal},\mathrm{product}}+m_{\mathrm{metal},\mathrm{liquid}\;\mathrm{fractions}}+m_{\mathrm{metal},\mathrm{solid}\;\mathrm{residues}}}{m_{\mathrm{metal},\mathrm{charged}}}\right]\times 100\%.$$

## 3. Polypyrrole as a Potentially Green Nanoplatform: Advantages and Intrinsic Limitations

### 3.1. Why PPy Can Provide a Greener Starting Point than Many Inorganic Nanomaterials

Polypyrrole (PPy) occupies a distinctive position within the broader landscape of photothermal nanomaterials because its optical functionality can be obtained from a conjugated organic polymer rather than from a metallic, metal-oxide, or semiconductor core. This compositional difference may reduce dependence on noble metals, rare-earth elements, and multicomponent inorganic heterostructures. However, the absence of a persistent inorganic core should be regarded as a favorable starting characteristic rather than direct evidence that a PPy formulation is environmentally preferable or biologically degradable.

For several inorganic nanoparticle systems, in vivo distribution and clearance are strongly influenced by core composition, particle size, aggregation state, and surface coating. For example, iron-oxide nanoparticles with different sizes and coatings accumulated predominantly in the liver and spleen, with substantial amounts of some PEGylated formulations remaining after two weeks [79]. Such observations demonstrate the potential for prolonged organ retention, but they do not justify treating all inorganic nanoparticles as equally persistent or toxic.

Conversely, polymeric composition alone does not guarantee rapid degradation or clearance. PPy contains a relatively stable conjugated backbone, and long-term experiments have demonstrated that PPy grains can retain their primary morphology in aqueous media for up to one year. However, dedoping and counterion release vary with pH and dopant identity [80]. Accordingly, PPy should not be described as intrinsically biodegradable without formulation-specific evidence. Its post-administration fate may instead involve combinations of dedoping, oxidation, fragmentation, coating degradation, disassembly, cellular sequestration, and size-dependent elimination. These processes must be demonstrated directly for the specific nanostructure being evaluated. Functionally, the conjugated and doped electronic structure of PPy produces broad visible and near-infrared absorption associated with delocalized electronic states, polarons, and bipolarons [80,81]. This enables photothermal conversion without requiring plasmonic noble-metal domains. A recent PPy-containing nanoplatform reported a photothermal conversion efficiency of 38% under 808 nm irradiation [81], while morphology-engineered ultrathin PPy nanosheets exhibited strong broadband absorption and efficient photothermal activity in the second NIR window [29]. These findings confirm that PPy can function as an effective organic photothermal component. Nevertheless, photothermal conversion efficiencies reported by different studies should not be directly ranked unless particle concentration, absorbance, laser wavelength, and power density, irradiation geometry, heat-loss assumptions, and calculation procedures are comparable.

The principal compositional advantage of PPy is therefore not that it universally outperforms gold or other inorganic photothermal agents. However, that clinically relevant optical absorption can be generated without making a metallic core indispensable. This may reduce material complexity and metal-associated persistence in appropriately designed formulations. However, these potential benefits can be diminished when PPy is combined with metal ions, inorganic imaging agents, persistent coatings, or multiple functional components. The sustainability of the final formulation must consequently be evaluated at the system level rather than inferred solely from the PPy component.

From a synthetic perspective, PPy is commonly produced by chemical oxidative polymerization, which can be carried out in aqueous or predominantly aqueous media at relatively low temperatures. Recent work has demonstrated controllable formation of PPy nanoparticles via low-temperature oxidative polymerization, with particle size and dispersibility varying with monomer, oxidant, and stabilizer concentrations [82]. Such conditions may avoid the elevated temperatures and pressures required by some solvothermal inorganic syntheses. However, reaction temperature and solvent identity alone are insufficient to establish a lower environmental burden.

For example, the same low-temperature PPy synthesis involved acidic conditions, an ice bath, nitrogen protection, freshly distilled pyrrole, precipitation in a large volume of cold methanol, repeated centrifugation and washing, and subsequent vacuum drying [82]. These downstream requirements illustrate why an apparently mild polymerization step should not automatically be classified as a green process. A defensible comparison with inorganic nanomaterial synthesis would require quantitative information on energy consumption, solvent and water use, isolated yield, monomer conversion, oxidant utilization, purification demand, waste treatment, and process mass intensity.

An important advantage of oxidative polymerization is that particle formation and functional integration can occur concurrently. Recent studies have used dopamine-assisted assembly to generate hybrid PPy–polydopamine nanosheets with tunable optical and imaging properties [83]. Surface morphology has also been modified during PPy nanoparticle formation using a removable micellar template, yielding rough particles with enhanced drug loading and photothermal performance compared with smoother PPy particles [84]. These findings support a synthesis–structure–function relationship in which templates, stabilizers, dopants, and polymerization conditions influence both morphology and therapeutic performance.

Nevertheless, such strategies should not automatically be described as “structure-as-function” green synthesis. Templates, surfactants, biological coatings, and functional molecules may introduce additional reagents, purification steps, batch variability, and uncertainties in biological fate. In situ or one-pot functionalization is environmentally advantageous only when the avoided post-synthetic steps outweigh the material and processing burden introduced by the integrated components.

PPy can provide a potentially less metal-intensive and comparatively mild starting platform for photothermal nanomedicine. Its organic conjugated backbone, intrinsic NIR absorption, compatibility with oxidative polymerization, and capacity to adjust morphology and function during synthesis are meaningful design advantages. However, these characteristics do not establish inherent sustainability. The environmental and translational performance of each PPy formulation ultimately depends on the complete synthesis route, auxiliary materials, purification requirements, residual species, structural stability, degradation products, biodistribution, and clearance.

### 3.2. Intrinsic Sustainability Bottlenecks of Pristine PPy

Despite its apparent advantages, pristine PPy is far from an ideal sustainable nanomaterial. A more critical examination reveals several structural and functional limitations that directly affect its long-term applicability in nanomedicine. Because pristine PPy does not itself contain abundant carboxyl groups, carbodiimide-mediated coupling generally requires the prior introduction of a carboxylated component or another reactive functional layer [85,86]. These additional functionalization and purification operations may partly offset the process advantages of the initial aqueous polymerization [85,86].Morphologically, PPy nanoparticles tend to adopt dense, solid structures with limited internal porosity [24,71,87]. While such compactness can be advantageous for structural stability, it becomes problematic with respect to degradation [71,80]. Restricted diffusion pathways hinder both chemical breakdown and physical disassembly, raising concerns about long-term persistence [71,80]. Restricted diffusion pathways hinder both chemical breakdown and physical disassembly, raising concerns about long-term persistence. Importantly, the frequently cited “biocompatibility” of PPy does not necessarily translate into safe clearance or environmentally benign end-of-life behavior [35,80,88].

Metal-free PPy is most commonly evaluated as a PTT-dominant material [24,29,87]. The principal limitations of PTT include finite NIR tissue penetration, heterogeneous heat distribution, heat dissipation via blood perfusion, difficulty controlling intratumoral thermal dose, potential injury to adjacent normal tissue, and heat-shock responses following sublethal heating [5,89]. Tumor hypoxia primarily restricts oxygen-dependent PDT rather than PTT itself [5,90]. Incorporating ROS-generating functionality may broaden the therapeutic mechanism, but the oxygen dependence and specificity of the resulting pathway must be evaluated separately.

**Figure 2:**
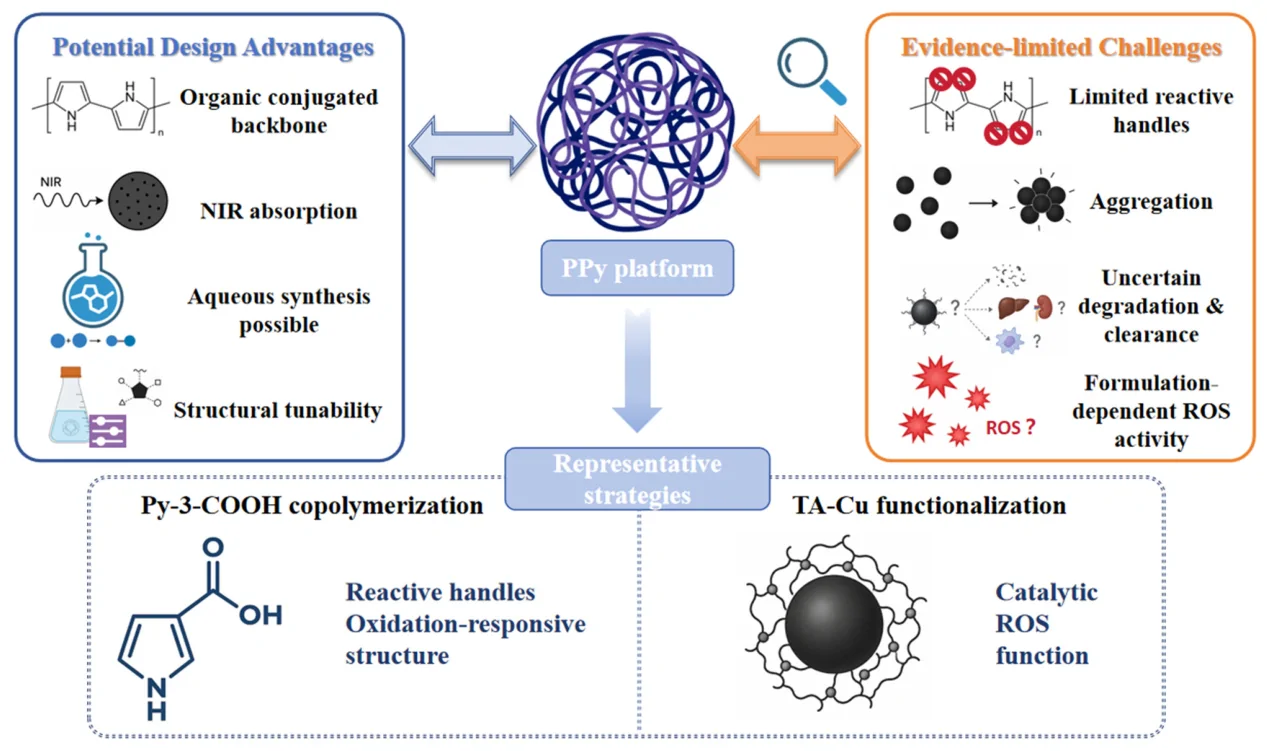
Design opportunities, evidence-limited challenges, and sustainability-guided strategies for PPy nanomedicines.

These observations indicate that the sustainability of PPy-based systems cannot be inferred solely from their composition or synthesis route but must be evaluated through a broader structure–function–performance framework [60,61]. As illustrated in Figure 2, the intrinsic advantages of PPy coexist with clear structural and functional bottlenecks, which, in turn, motivate targeted engineering strategies to improving degradability, functionality, and overall material efficiency [60,61].

### 3.3. Py-3-COOH–Derived PPy: Degradability as a Structure-Driven Outcome

The introduction of carboxyl-functionalized monomers such as Py-3-COOH represents a rational attempt to address multiple limitations simultaneously [71,91]. Rather than treating degradability as an empirical observation, it is more instructive to interpret it as a direct consequence of structural design [91]. A key issue in current literature is the reliance on indirect indicators of degradation [92,93]. For instance, attenuation of NIR absorption or photothermal performance under oxidative conditions is often used as evidence of material breakdown [71]. While such changes do suggest disruption of the conjugated system, they do not necessarily confirm polymer fragmentation, mineralization, or biological clearance [92,93]. From a methodological standpoint, this distinction is non-trivial.

A more rigorous evaluation requires a multi-layered evidence chain [92,93,94]. Techniques such as gel permeation chromatography (GPC) can reveal reductions in molecular weight, while LC–MS enables identification of low-molecular-weight degradation products [92,93]. Total organic carbon (TOC) analysis provides insight into carbon conversion, and transmission electron microscopy (TEM) can visualize structural collapse [93]. For systems involving metal components, inductively coupled plasma mass spectrometry (ICP-MS) should be incorporated to track ion release [94]. Only through such combined approaches can degradability be elevated from a qualitative claim to a verifiable property [92,93,94].

Beyond degradation, the formation of hollow structures introduces an additional dimension of sustainability [95]. Hollow PPy nanospheres inherently possess higher surface-to-volume ratios, which increases the accessibility of active sites [71,95]. In practical terms, this can translate into a reduced material dosage to achieve comparable therapeutic effects. From a green chemistry perspective, this reflects an improvement in functional efficiency—delivering more output per unit material input [37,43,95]. Equally important is the synthetic route itself. The use of Py-3-COOH enables the incorporation of functional groups incorporation during polymerization, eliminating the need for post-synthetic coupling steps [91]. This not only simplifies the process but also reduces reliance on auxiliary reagents and solvents [37,43,91]. In doing so, it preserves the advantages of aqueous-phase synthesis while enhancing structural versatility. In this sense, the Py-3-COOH strategy exemplifies a broader principle: sustainability in nanomaterials is often achieved not through additional complexity, but through more intelligent integration of structure and function at the earliest stages of design [37,38,43].

### 3.4. TA–Cu Metal–Polyphenol Networks: ROS Enhancement Under Controlled Trade-Offs

The incorporation of tannic acid–copper (TA–Cu) metal–polyphenol networks introduces a distinct design logic—one that prioritizes functional enhancement through controlled redox activity [96,97]. However, this approach is inherently accompanied by trade-offs that must be explicitly acknowledged. Experimental observations consistently indicate that TA–Cu coating enhances ROS generation, thereby enabling photodynamic or chemodynamic therapeutic effects [96,97]. At the same time, increasing coating thickness tends to reduce photothermal conversion efficiency and promote particle growth and aggregation [98]. These effects are not incidental but arise from fundamental physicochemical interactions, including optical attenuation, increased scattering, and changes in surface charge [98]. From a design perspective, the challenge is therefore not simply to maximize ROS output, but to identify an optimal balance [96,97]. Thin, self-limiting coatings—achieved through controlled ligand concentration, short coordination times, or competitive binding strategies—may provide a practical pathway toward this balance [98,99]. Such approaches aim to retain photothermal performance while introducing sufficient redox activity [71,96,98].

Another critical consideration is metal stability. TA–Cu systems are susceptible to ion exchange in biologically relevant environments, raising concerns about copper ion release and associated cytotoxicity [94,96,97]. Quantitative leaching studies under conditions such as phosphate-buffered saline (PBS), serum, or glutathione-rich media should be considered essential, rather than optional [60,94,97]. Importantly, these data should be correlated with biological outcomes to establish a meaningful risk–benefit relationship [60,94,97]. In addition, ROS characterization requires greater specificity [100,101]. Common colorimetric assays, while convenient, often fail to distinguish between different ROS species. Techniques such as electron paramagnetic resonance (EPR) spectroscopy, or the use of selective probes (e.g., SOSG for singlet oxygen, HPF for hydroxyl radicals), are necessary to achieve mechanistic clarity. Without such differentiation, claims of “enhanced ROS” remain overly generalized [100,101]. It is worth noting that tannic acid itself represents a favorable component from a sustainability standpoint [37,38,97,99]. As a naturally derived polyphenol, it has gained increasing attention in biomaterials research, particularly in the context of metal–polyphenol networks [97,99]. Its inclusion can therefore strengthen the overall green narrative—provided that associated risks are properly managed [37,38,94,97]. Ultimately, TA–Cu integration should not be viewed as a straightforward performance upgrade but rather as a trade-off-driven design strategy [94,96,97]. Recognizing and quantifying these trade-offs is essential for advancing PPy-based systems toward genuinely sustainable nanomedicine [37,60,71].

## 4. Green Synthetic and Functional Design Across PPy Polymerization Routes: Evidence and Trade-Offs

### 4.1. Coupling Synthesis Variables with Functional Outcomes

Oxidative polymerization is the stage at which the process characteristics and biomedical functions of polypyrrole (PPy) become closely coupled [102,103,104]. The oxidant or initiating system affects not only pyrrole conversion but also particle nucleation, conjugation length, oxidation state, heteroatom or metal incorporation, surface functionality, and the subsequent requirements for purification and surface modification [105,106,107]. These changes can influence colloidal size, optical absorption, photothermal conversion, catalytic activity, oxidative responsiveness, and biological interactions [24,55,95,103,104,108].

However, published PPy studies differ substantially in monomer concentration, oxidant-to-monomer ratio, stabilizer, temperature, reaction time, particle architecture, irradiation wavelength, laser power density, and the method used to calculate photothermal conversion efficiency (PCE) [24,55,75,103,107,109]. Therefore, cross-study numerical differences should not be interpreted as proof that the oxidant alone causes a particular functional or sustainability outcome [75,104].

To avoid conflating controlled evidence with indirect cross-study comparison, this section distinguishes three levels of evidence, consistent with established principles for the narrative synthesis of heterogeneous quantitative studies.

1. Controlled within-study comparisons, in which one or more synthetic variables were deliberately changed while other conditions were substantially maintained [103,104];
2. Direct formulation-specific measurements, including particle size, temperature elevation, PCE, optical attenuation, or catalytic assay results measured for the reported formulation [75,109];
3. Cross-study benchmarks, which are retained to define experimentally reported performance ranges, but are not treated as properties of the target formulation [110].

This evidence structure allows quantitative results to remain informative without converting heterogeneous literature data into an unvalidated greenness ranking.

**Figure 3:**
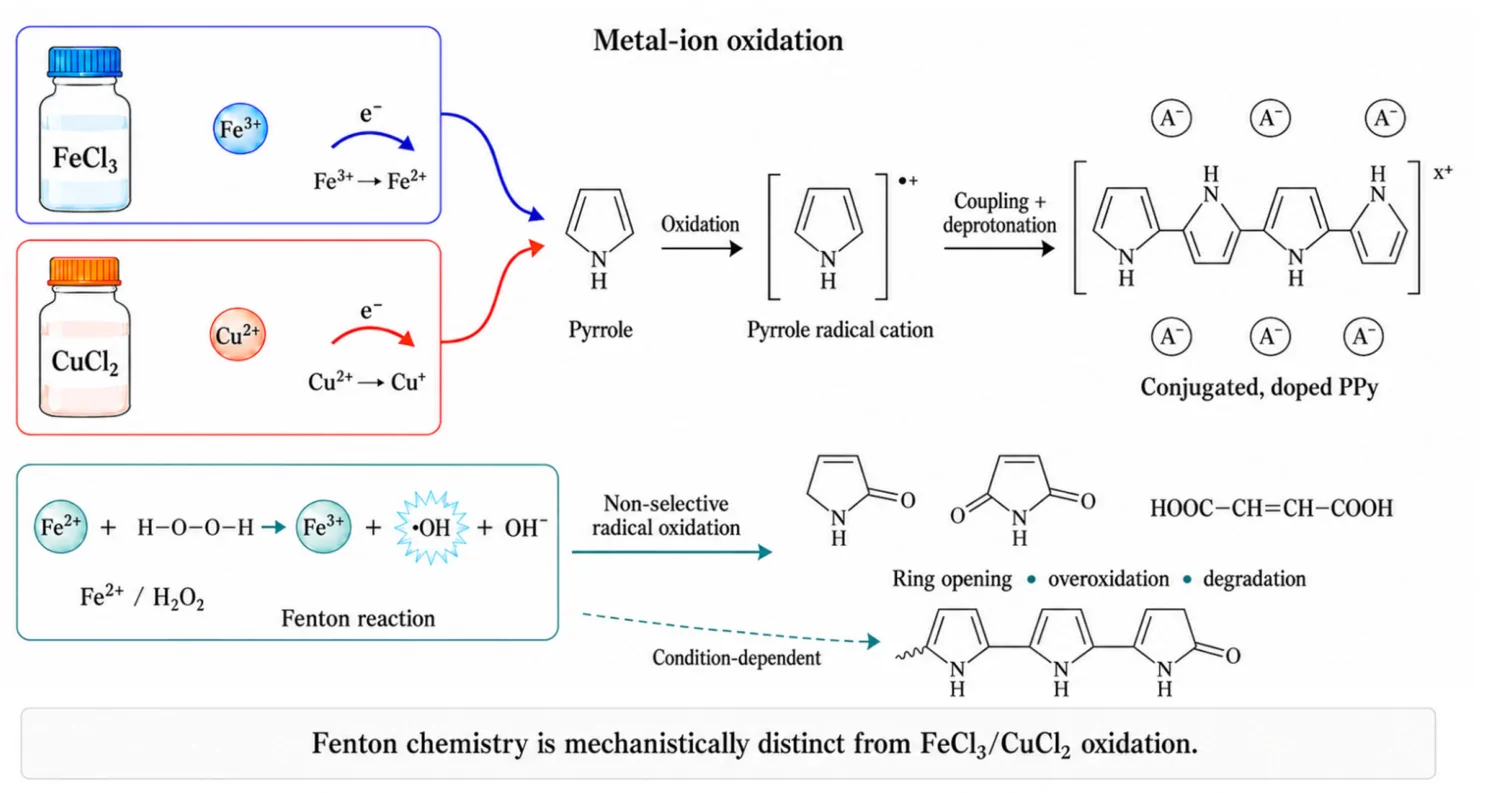
Proposed oxidant-mediated pathways for PPy formation and Fenton-driven oxidative transformation. FeCl_3_- and CuCl_2_-associated oxidation is depicted through a representative pyrrole radical-cation pathway leading to coupling, deprotonation, and doped PPy formation; this pathway is not presented as exclusive. The Fe^2+^/H_2_O_2_ branch is separated to show that Fenton-derived radicals may also promote non-selective oxidation, ring opening, overoxidation, or degradation, while oxidized or defect-containing PPy may form under some conditions. The structures are illustrative and do not identify a unique mechanism or product distribution.

The mechanistic distinction among the three initiating systems is summarized schematically in Figure 3. FeCl_3_ and CuCl_2_ are represented by a simplified metal-ion-mediated oxidation and coupling pathway; however, oxidized oligomer–monomer reactions may also contribute, and the dominant propagation pathway depends on the complete reaction conditions [70]. The Fe^2+^/H_2_O_2_ branch is shown separately because Fenton-derived radicals can also promote non-selective oxidation and overoxidation, while peroxide-mediated PPy formation remains condition-dependent [111,112]. Accordingly, the diagram presents proposed pathways rather than a unique mechanism.

### 4.2. FeCl_3_-Mediated Polymerization: Process Simplicity and Photothermal-Oriented Design

#### 4.2.1. Synthetic Route and Reaction Conditions

FeCl_3_-mediated oxidative polymerization remains one of the most frequently used routes for preparing PPy because polymerization can be conducted in aqueous media at or near room temperature [28]. Stabilizers such as poly (vinyl alcohol), poly (vinylpyrrolidone), surfactants, or hydrophilic polymers are commonly introduced to regulate particle nucleation and prevent aggregation [102]. Nevertheless, monomer conversion and particle characteristics remain sensitive to temperature, FeCl_3_ concentration, mixing, reaction time, and the presence of dopants [103].

Brusamarello et al. conducted a controlled investigation of FeCl_3_·6H_2_O-mediated pyrrole polymerization and reported pyrrole conversions ranging from 67.77% to 89.83% after 240 min under the examined conditions. A conversion close to 90% was obtained at 20 °C using 0.5 mol L^−1^ FeCl_3_·6H_2_O. The average particle size decreased from approximately 360 nm at 5 °C to 320 nm at 20 °C, while sodium dodecyl sulfate-containing PPy reached an electrical conductivity of 13.30 S cm^−1^ [103]. These data provide controlled evidence that reaction conditions and dopant selection affect conversion, particle formation, and bulk electrical properties. However, the particles were not developed as an injectable nanomedicine, and conductivity should not be used as a direct surrogate for photothermal or biological performance [24].

A complementary process benchmark was reported by Seike et al., who prepared PPy by mechanically mixing pyrrole with solid FeCl_3_ under nitrogen without a polymerization solvent. After purification, a PPy yield of 74% was obtained. The product consisted mainly of grains with dimensions in the tens-of-micrometers range rather than biomedical nanoparticles [24]. This example demonstrates an important trade-off: eliminating the reaction solvent can reduce one material input, but it does not automatically ensure the particle size, dispersibility, sterility, or surface characteristics required for nanomedicine [24,59,69]. Furthermore, the use of solvents during washing and purification remains relevant when evaluating the overall process [19].

Accordingly, the FeCl_3_ route can be considered experimentally accessible and potentially process-efficient [28,46]. However, it should not be described as intrinsically benign or low risk without reporting monomer conversion, isolated yield, washing inputs, residual iron, and colloidal quality [59].

#### 4.2.2. Photothermal Performance and Quantitative Benchmarks

FeCl_3_-derived PPy nanoparticles provided some of the earliest quantitative evidence supporting PPy as an organic photothermal agent. Chen et al. prepared sub−100-nm PPy nanoparticles with an average diameter of approximately 46 nm and measured a photothermal conversion efficiency (PCE) of approximately 44.7% under 808-nm irradiation [24]. The nanoparticles exhibited concentration-dependent photothermal heating and enabled photothermal ablation in both cellular and animal tumor models. However, this value represents a formulation- and protocol-specific measurement rather than a universal PCE for FeCl_3_-derived PPy [75].

Higher PCE values have subsequently been reported by modifying PPy morphology and extending its optical response into the second near-infrared window. Wang et al. prepared ultrathin PPy nanosheets with lateral dimensions of approximately 100 nm and a thickness of approximately 2 nm [29]. The nanosheets exhibited PCE values of 55.6% at 808 nm and 64.6% at 1064 nm, together with a mass extinction coefficient of 27.8 L g^−1^ cm^−1^ at 1064 nm. However, their preparation relied on an FeOCl-based space-confined template, followed by acid treatment to remove it. The resulting optical improvement should therefore be evaluated together with the additional template synthesis, acid washing, purification, and associated material inputs [19].

Zeng et al. reported ultrasmall PPy–PEG nanoparticles with dimensions of approximately 2 nm [35]. The measured PCE was 33.35% at 808 nm and 41.97% at 1064 nm. The reported photothermal experiments included irradiation at 1.0 W cm^−2^, and the formulation retained a reproducible photothermal response during repeated heating–cooling testing. Although its PCE was lower than that of the ultrathin nanosheets, the ultrasmall dimensions and PEG-containing surface were designed to promote aqueous dispersion, tumor accumulation, and renal clearance.

Together, these studies define an experimentally reported PPy PCE range of approximately 33.35–64.6% across substantially different particle sizes, morphologies, surface chemistries, irradiation wavelengths, and measurement protocols. This range is retained only as a cross-study performance benchmark. It should not be assigned to PPy@PyCOOH or TA–Cu-coated PPy@PyCOOH unless PCE is measured directly for those formulations using a clearly reported energy-balance procedure [24,29,35].

#### 4.2.3. Functional Profile and Limitations

The main functional advantage of conventional FeCl_3_-mediated PPy is its broad optical absorption, including strong near-infrared absorption, together with efficient conversion of absorbed light into heat via predominantly nonradiative relaxation pathways [24,29]. It is therefore particularly suitable for formulations in which photothermal heating is designed as the principal therapeutic mechanism.

However, the route also presents unresolved process and analytical issues. Iron-containing residues or iron-derived species may remain associated with FeCl_3_-polymerized PPy after synthesis, depending on the polymerization, washing, and purification conditions [113]. The magnitude and chemical form of such residual iron cannot be inferred reliably from the initial FeCl_3_ feed because only part of the oxidant may become associated with the isolated polymer. At the same time, the remainder is removed in the reaction medium and washing fractions [69]. Residual iron should therefore be quantified directly using an appropriate elemental analysis method, such as inductively coupled plasma optical emission spectroscopy or inductively coupled plasma mass spectrometry and reported together with the washing procedure and relevant sample preparation details [59].

Similarly, the absence of a deliberately incorporated catalytic metal does not demonstrate that FeCl_3_-derived PPy has zero ROS activity. It indicates only that catalytic ROS generation has not been established as an intentionally engineered function unless it is demonstrated using formulation-specific ROS assays and appropriate controls [114]. Claims such as “negligible ROS generation”, “low biological risk”, or “high structural stability” should therefore be supported by measurements performed on the specific formulation under the relevant irradiation, medium, dose, and exposure conditions rather than assigned to the FeCl_3_ route as general material properties.

### 4.3. CuCl_2_-Mediated Polymerization: One-Step Redox Functionalization and Metal-Management Trade-Offs

#### 4.3.1. Synthetic Route and Mechanistic Distinction

CuCl_2_-mediated polymerization can simultaneously oxidize pyrrole and introduce copper-associated enzyme-mimetic redox functionality. This approach should be distinguished from two related but non-equivalent strategies: post-synthetic adsorption or coordination of copper onto preformed PPy, and deposition of a tannic acid–copper coating onto a preformed PPy core [71,115]. These routes differ in the stage at which copper is introduced, its probable coordination environment, the required purification procedures, and the potential pathways for copper release.

Zeng et al. used CuCl_2_ in a one-step oxidative polymerization of pyrrole to produce Cu-doped PPy nanoparticles denoted CuP [115]. The resulting CuP was subsequently PEGylated to form CuPP, and the incorporation of copper was confirmed by elemental mapping and Cu 2p X-ray photoelectron spectroscopy. Dynamic light scattering confirmed nanoscale size distributions for CuP and CuPP, and CuPP remained visibly dispersible in water, phosphate-buffered saline, and cell culture medium after one week. This study provides direct evidence for one-step formation of a copper-containing PPy nanozyme; its synthesis and catalytic data should not be conflated with those obtained from post-synthetic TA–Cu coatings [71,115].

#### 4.3.2. Photothermal and Catalytic Performance

The CuP nanoparticles exhibited a broad absorption band in the 1000–1100-nm region. At a CuP concentration of 20 μg mL^−1^, irradiation at 1064 nm and 0.5 W cm^−2^ for 5 min increased the dispersion temperature to approximately 46 °C [116]. The photothermal response remained largely unchanged over four heating–cooling cycles under the same irradiation conditions.

The same formulation exhibited peroxidase-like catalytic activity. In an H_2_O_2_-containing methylene-blue degradation assay, approximately 50% MB depletion was observed within 20 min at 45 °C [116]. Time-dependent glutathione consumption was also observed using a DTNB-based assay and was attributed by the authors to a redox reaction between GSH and Cu^2+^-containing active sites in CuP. Together, these measurements support the coupling of copper-associated redox activity, thiol depletion, and H_2_O_2_-dependent radical generation [115,117]. However, MB depletion is an assay-specific indicator of oxidation and should not be interpreted as a direct or absolute measure of hydroxyl radical yield [117,118].

Related one-step Cu-doped PPy nanozymes have also been reported by Zeng et al., with catalase-, glutathione peroxidase-, and peroxidase-like activities [115], and by Chen et al., with Cu-mediated conversion of H_2_O_2_ into O_2_ and ·OH, Cu(II)/Cu(I)-associated GSH depletion, and ultrasound-enhanced nanocatalytic therapy [117]. Collectively, these studies support a route–function association between copper incorporation and multifunctional redox activity in the investigated Cu-doped PPy formulations [115,116,117]. They do not, however, establish that every CuCl_2_-derived PPy formulation has the same catalytic rate, copper speciation, ROS output, photothermal response, or biological safety profile [118].

#### 4.3.3. Sustainability and Translation Considerations

Copper incorporation can integrate enzyme-mimetic redox activity into the PPy formulation. It may therefore avoid the need to introduce a separate enzyme or catalytic nanoparticle when such activity is required [115,116]. Nevertheless, the nominal CuCl_2_ feed is not equivalent to the copper content retained in the purified formulation [59,119]. A defensible assessment should include direct measurement of total copper content, copper oxidation state, batch-to-batch variability, and copper release under relevant exposure conditions [59,119,120]. Release testing should preferably include water or a formulation buffer, a serum-containing medium, an acidic lysosomal-mimicking medium, and other biological fluids relevant to the intended route of administration, because copper dissolution and ligand-assisted release can vary substantially with pH and medium composition [120,121].

The CuP-loaded hydrogel study reported no marked loss of body weight and no obvious histopathological abnormalities in the examined major organs during the short in vivo treatment period [116]. This provides useful formulation- and protocol-specific evidence of short-term tolerability, but it does not establish chronic safety, systemic clearance, or the general biocompatibility of Cu-doped PPy formulations [116,122]. Green synthesis, short-term tolerability, and clinical translatability should therefore be treated as separate evaluation dimensions [122,123].

CuCl_2_-mediated polymerization should consequently be regarded as a function-integrating route whose value depends on whether copper-associated catalytic activity provides a measurable therapeutic benefit relative to the additional requirements for metal-content characterization, reproducible manufacturing, and release control [59,115,116,119]. It should not be assigned a universally higher or lower sustainability position than FeCl_3_-mediated polymerization without formulation-specific comparisons using equivalent process boundaries, functional units, and performance criteria [123].

### 4.4. Fe^2+^/H_2_O_2_-Mediated Polymerization: Oxidation-Responsive Functionalization and Evidence Boundaries

#### 4.4.1. Synthetic Route and Formulation-Specific Inputs

Fe^2+^/H_2_O_2_-mediated polymerization uses in situ radical generation to initiate oxidative polymerization of pyrrole and can be combined with functional pyrrole comonomers. In the PPy@PyCOOH formulation reported by Du et al., 33.3 mg of pyrrole-3-carboxylic acid (Py-3-COOH) and 1.2 mg of NaOH were dissolved in 5 mL of deionized water. Subsequently, 0.5 mL of FeCl_2_·4H_2_O solution at 26 mg mL^−1^, 83 μL of pyrrole, and 300 μL of 30 wt% H_2_O_2_ were introduced, and the reaction proceeded for 3 h at room temperature [71].

The resulting PPy@PyCOOH particles had a reported hydrodynamic diameter of 248 ± 12 nm, whereas transmission electron microscopy revealed hollow nanospheres with a characteristic diameter of approximately 100 nm [71]. The difference between the DLS and TEM values may reflect the hydrated and potentially associated state measured by DLS, the dry-state dimensions visualized by TEM, and the different weighting and measurement principles of the two techniques [124]. Both measurements should therefore be retained, along with their analytical methods and dispersion conditions, rather than collapsed into a single particle-size value [125].

The route introduces carboxyl-containing pyrrole units during polymer formation and thus avoids a separate post-synthetic carboxylation step for the PPy@PyCOOH core. However, the term “one-step synthesis” applies only to the formation of the carboxyl-functionalized PPy core. Preparation of the complete TA–Cu-coated formulation requires a subsequent tannic acid–Cu^2+^ coordination step and should therefore not be described as a one-pot or single-step synthesis [71].

#### 4.4.2. Oxidative Optical Attenuation, Photothermal Response, and ROS Output

Under an accelerated oxidative condition containing 10 mM H_2_O_2_ at pH 6.5, PPy@PyCOOH exhibited an 82.5 ± 4.1% decrease in absorbance at 808 nm after 60 min, compared with a 17.8 ± 2.6% decrease for pristine PPy [71]. This controlled within-study comparison supports the conclusion that incorporation of PyCOOH increased the susceptibility of the PPy optical structure to oxidation under the tested conditions.

Nevertheless, a loss of absorbance at a single wavelength provides evidence of optical attenuation and possible disruption of the conjugated polymer structure. However, it does not, by itself, demonstrate complete chain degradation or chemical mineralization [126]. It also does not establish renal clearance or in vivo biodegradation, which would require direct characterization of degradation products, mass balance, pharmacokinetics, biodistribution, and excretion [127]. Moreover, 10 mM H_2_O_2_ should be identified as an accelerated oxidative challenge rather than presented as a representative or universal tumor H_2_O_2_ concentration, because commonly cited tumor-microenvironment H_2_O_2_ levels are generally in the micromolar rather than millimolar range [128].

For the same formulation family, PPy@PyCOOH at 100 μg mL^−1^ reached approximately 34 °C after 5 min of 808-nm irradiation at 1.0 W cm^−2^, whereas TA–Cu-coated PPy@PyCOOH reached approximately 30–31 °C under matched conditions. Both formulations exhibited broadly reproducible heating profiles over three irradiation cycles [71]. These direct formulation-specific data indicate that addition of the TA–Cu layer did not increase the measured bulk temperature elevation under the reported conditions, despite introducing additional copper-associated catalytic functionality. However, the endpoint temperatures should not be treated as direct measurements of PCE unless a complete energy-balance analysis is performed [75].

The TA–Cu-coated formulation generated a ROS-probe fluorescence response of 1.52 ± 0.08-fold relative to the control in the reported assay [71]. This result should be described as an assay-specific relative signal rather than an absolute ROS generation rate or molar radical yield, because fluorescence-based ROS probes are influenced by probe selectivity, reaction kinetics, optical interference, and assay conditions [129]. Because the ROS assay, oxidative optical-attenuation experiment, and photothermal experiment quantify distinct phenomena, their numerical outputs should be reported separately rather than combined into a single composite performance score [75,129].

#### 4.4.3. Sustainability Assessment

From a green chemistry perspective, the Fe^2+^/H_2_O_2_ route is noteworthy because it uses an aqueous peroxide-based initiating system and assigns the iron salt primarily a polymerization-mediating role rather than deliberately incorporating it as a permanent therapeutic payload. The broader feasibility of peroxide-mediated PPy formation is supported by earlier studies in which pyrrole was polymerized using H_2_O_2_ with a catalytic amount of iron in acidified aqueous medium or using H_2_O_2_ together with a surfactant to form PPy nanoparticles [111,112]. These studies establish the general feasibility of iron-catalyzed or H_2_O_2_-initiated PPy formation, whereas the more recent PPy@PyCOOH formulation extends this chemistry to carboxyl-functionalized hollow particles with oxidation-responsive optical behavior [71]. Nevertheless, aqueous processing and mild reaction temperatures alone do not confer superior sustainability, because sustainable nanomaterial design requires the combined consideration of functional performance, resource consumption, process efficiency, and material-associated hazards [59,130].

Nevertheless, the E-factor nor process mass intensity (PMI) has been reported for this formulation. PMI is particularly relevant because it accounts for the total mass of reagents, solvents, water, and other materials used throughout reaction and work-up [16]. Consequently, repeated centrifugation and washing, dilute processing conditions, unrecovered material in supernatants, and the energy required for separation cannot be disregarded merely because water is used as the polymerization medium [16,59]. A quantitative process claim would require, at a minimum, pyrrole conversion, isolated particle yield, total washing-water volume, solids concentration, separation requirements, and directly measured residual iron. Until these data are available, the route can reasonably be described as process-simplifying, but not as demonstrably low-waste.

The oxidative-response evidence also requires a narrower interpretation. PPy@PyCOOH exhibited an 82.5 ± 4.1% decrease in absorbance at 808 nm after 60 min in 10 mM H_2_O_2_ at pH 6.5 [71]. This is strong evidence of rapid optical attenuation under accelerated oxidative stress, but it does not demonstrate complete depolymerization, mineralization, or biological clearance [18]. Biomedical literature commonly describes tumor-associated H_2_O_2_ concentrations in the micromolar range, although the values are spatially and temporally heterogeneous and remain difficult to quantify reliably in vivo [128,131]. The 10 mM condition should therefore be identified as an accelerated oxidative challenge rather than a physiologically representative tumor-mimicking environment.

Moreover, absorbance loss alone cannot distinguish de-doping or overoxidation of the conjugated backbone from chain scission, particle fragmentation, dissolution, or mineralization. Current biomaterial degradation methodology recommends combining physical and morphological observations with chemical characterization of the fragmented material and the resulting products [18]. For PPy@PyCOOH, a more complete assessment would include time-resolved DLS and TEM, FTIR or XPS analysis of backbone oxidation, total organic carbon measurements, and LC–HRMS or size-exclusion analysis where analytically feasible. Total and leachable iron should also be quantified using ICP-MS or ICP-OES, and residual H_2_O_2_-dependent catalytic activity should be examined in serum-containing and lysosomal-mimicking media. The use of iron primarily as a polymerization mediator does not itself demonstrate its complete absence from the purified particles [59,130].

Overall, the Fe^2+^/H_2_O_2_ route remains a conceptually important sustainability-guided design strategy because core formation, carboxyl functionalization, and oxidation responsiveness are achieved in a single polymerization stage [71]. This integration may avoid an additional post-synthetic carboxylation step. Its proposed sustainability advantage should now be tested through measurable criteria—including high conversion and isolated yield, reduced PMI and washing demand, controlled residual iron, responsiveness under physiologically relevant oxidant exposure, and identification and safety evaluation of the resulting transformation products—rather than inferred from aqueous synthesis alone [16,18,59,130].

### 4.5. Dual-Axis Comparison of Green Synthesis and Functional Evidence

When considered collectively, these three oxidant systems should be treated as context-dependent design options with different evidence-supported trade-offs in structural stability, functional complexity, process burden, and biological safety, rather than being positioned on a single sustainability continuum. To preserve quantitative context without conflating formulation-specific measurements with cross-study estimates, Table 3 separates directly reported data for PPy@PyCOOH and TA–Cu-coated PPy@PyCOOH from quantitative benchmarks reported for chemically related but non-identical systems. Literature benchmarks are provided only to illustrate the range attainable under specified experimental conditions and are not assigned to the target formulations. Parameters that cannot be calculated from the available material-balance or calorimetric data are designated as not reported (NR).

### 4.6. Sustainability-Guided Design Implications

The available evidence does not justify a universal sustainability ranking of FeCl_3_-, CuCl_2_-, and Fe^2+^/H_2_O_2_-mediated polymerization routes. Sustainable nanomaterial selection requires that functional performance be evaluated alongside process inputs, material-associated hazards, stability, and downstream biological or environmental fate [130,136]. Route selection should therefore balance the intended therapeutic function against the additional synthesis, purification, characterization, and safety requirements needed to achieve that function (Table 3).

For applications in which photothermal heating is the principal therapeutic mechanism, aqueous FeCl_3_-mediated polymerization provides a comparatively direct and experimentally established starting point. FeCl_3_-derived PPy nanoparticles with an average diameter of approximately 46 nm exhibited a reported photothermal conversion efficiency (PCE) of 44.7% at 808 nm. In comparison, morphology-engineered ultrathin PPy nanosheets reached PCE values of 55.6% at 808 nm and 64.6% at 1064 nm [24,29]. These results demonstrate that PPy can support efficient photothermal conversion, but they do not establish a low environmental burden or a favorable safety profile for the corresponding synthesis route. Monomer conversion, isolated yield, washing demand, residual iron, colloidal stability, degradation, biodistribution, and long-term fate remain separate evaluation parameters [59,130,136].

When catalytic ROS generation, glutathione depletion, or hypoxia modulation is required, CuCl_2_-mediated polymerization or related Cu-doped PPy designs can integrate photothermal and enzyme-mimetic redox functions within a single nanoformulation [115,116]. In the investigated systems, copper incorporation supported H_2_O_2_-dependent catalytic activity, glutathione consumption, oxygen generation, and multifunctional catalase-, glutathione peroxidase-, or peroxidase-like behavior [116]. However, this functional integration introduces additional requirements for the direct measurement of copper loading, oxidation state, or local chemical environment; batch reproducibility; ion release; biodistribution; and clearance [59,136]. The catalytic performance and safety profile of one Cu-doped PPy formulation should therefore not be generalized to all CuCl_2_-derived materials.

Fe^2+^/H_2_O_2_-mediated copolymerization offers a distinct design strategy by introducing carboxyl functionality and increasing oxidative susceptibility during the formation of the PPy core. Under an accelerated condition containing 10 mM H_2_O_2_ at pH 6.5, PPy@PyCOOH exhibited an 82.5 ± 4.1% decrease in absorbance at 808 nm after 60 min, compared with 17.8 ± 2.6% for pristine PPy under matched conditions [71]. This controlled comparison supports enhanced oxidative optical attenuation, but absorbance loss alone does not indicate complete degradation of the polymer chain, mineralization, biodegradation, or biological clearance [18]. The subsequent TA–Cu modification introduced an additional ROS-related response but also required formaldehyde, ethanol, ammonia, a copper precursor, an extended coating stage, and hydrothermal treatment. The complete formulation, therefore, illustrates functional gain accompanied by increased process complexity rather than an unambiguously greener route.

Comparison with dopamine–melanin and other organic photothermal materials further demonstrates why high PCE or nominally organic composition should not be used as stand-alone evidence of sustainability. Dopamine–melanin nanospheres have achieved a reported PCE of approximately 40% and are effective in vivo for photothermal treatment. However, their overall sustainability would still depend on monomer sourcing, solvent and reagent use, purification, formulation stability, biological transformation, and clearance [130,137]. Organic composition may reduce reliance on persistent inorganic components, but it does not, by itself, establish a low process burden, complete biodegradability, or low biological risk.

Overall, PPy polymerization routes should be selected through matched evaluation of therapeutic performance, process complexity, material composition, residual reagents and metals, colloidal and chemical stability, degradation products, biodistribution, and clearance [59,130,136]. FeCl_3_-mediated polymerization is most directly supported for photothermal-oriented design; Cu-containing routes provide stronger justification when catalytic redox functions produce a measurable therapeutic advantage; and Fe^2+^/H_2_O_2_-mediated copolymerization offers a promising route for integrating surface functionality and oxidation responsiveness during core formation. These are context-dependent design positions rather than a universal sustainability hierarchy.

## 5. Functionalization Strategies Under a Green Chemistry Lens: From Modification to Process–Efficiency Design

Functionalization of polypyrrole (PPy) nanomaterials is often framed as a downstream modification step aimed at compensating for intrinsic material limitations. However, once sustainability and translational feasibility are introduced as core evaluation criteria, this framing becomes insufficient. Functionalization does not occur independently of the synthetic pathway. Each added functionalization, purification, or washing operation can increase material inputs, solvent or water consumption, waste generation, and cumulative yield loss. Therefore, evaluation should extend beyond biological performance to include process-level factors such as route complexity, isolated yield, mass utilization, solvent and water consumption, and scalability [16,66]. This section re-examines two linked functionalization stages in a representative PPy system—(i) carboxyl-functionalized copolymerization used to prepare hollow PPy@PyCOOH nanospheres and (ii) subsequent tannic acid–copper (TA–Cu) metal–polyphenol network (MPN) coordination—as process-integrated design choices rather than solely as structural or functional upgrades [71]. The central question is therefore not only what these strategies achieve functionally, but also what additional material and processing burdens accompany those gains.

### 5.1. Carboxyl-Functionalized Copolymerization: Integrating Functionality During Polymer Formationon

Copolymerizing pyrrole with a carboxyl-bearing pyrrole comonomer can introduce carboxyl functionality during polymer formation rather than through a separate post-polymerization carboxylation step [138]. In related Py/Py-COOH systems, the incorporated carboxyl content and resulting material properties depend on the comonomer feed and polymerization conditions [139]. Once incorporated, the carboxyl groups can be activated by EDC/NHS for the covalent attachment of primary amine-containing molecules; EDC/NHS therefore represents subsequent activation and conjugation chemistry rather than the step that introduces carboxyl functionality [86,138].

In the representative PPy@PyCOOH system, hollow carboxyl-functionalized nanospheres were prepared through a single aqueous Fe^2+^/H_2_O_2_ oxidative copolymerization step that combined particle formation with carboxyl-functional-group incorporation [71]. This integration eliminates the need for a separate carboxyl introduction step but does not eliminate centrifugation, washing, or subsequent functionalization steps. Step integration alone does not establish a lower E-factor, process mass intensity, or overall process burden because mass-based metrics require defined system boundaries and measured material inputs and outputs [63]. Atom economy is a stoichiometric metric and should not be equated with isolated yield or whole-process mass efficiency [63,67].

#### 5.1.1. What the Literature Actually Reports

Roy et al. used FT-IR and XPS to demonstrate that the incorporation of Py-COOH into electrosynthesized Py/Py-COOH copolymers varied with applied potential, comonomer composition, and film-formation conditions [138]. In a related chemically polymerized system using 1-(2-carboxyethyl)pyrrole rather than Py-3-COOH, the feed rates of the functional monomer and oxidant, as well as the reaction time, affected surface functionality, conductivity, and overall yield [139]. These findings demonstrate that nominal comonomer feed should not automatically be equated with the composition or properties of the isolated copolymer.

For the Fe^2+^/H_2_O_2_-mediated PPy@PyCOOH route, reagent inputs and reaction conditions were described. However, monomer conversion, incorporated comonomer fraction, isolated dry yield, washing-water consumption, E-factor, and process mass intensity were not reported [71]. Because these values are absent from the primary study, they should be designated as not reported (NR) rather than estimated, and the available evidence should be interpreted as demonstrating route feasibility rather than a quantitative process-efficiency advantage [63,71].

#### 5.1.2. Hollow Structures: Morphological Evidence and Unresolved Efficiency Gains

Du et al. observed hollow PPy@PyCOOH nanospheres with an overall size of approximately 100 nm and shell thicknesses of 10–20 nm and proposed in situ nanobubble templating as a plausible mechanism for their formation. However, this mechanism was not directly demonstrated [71]. More generally, hollow nanostructures can provide internal void volume, high surface-to-volume ratios, and additional space for molecular loading, although these characteristics remain dependent on shell structure and accessibility [140]. Du et al. did not report specific surface area or provide a mass-normalized comparison with a compositionally matched dense PPy@PyCOOH control; therefore, a morphology-derived efficiency gain cannot yet be quantified [71].

At 100 μg mL^−1^, PPy@PyCOOH dispersions reached approximately 34 °C after 5 min of 808 nm irradiation at 1 W cm^−2^. They showed reproducible heating over three on/off irradiation cycles, but neither the photothermal conversion efficiency nor the mass concentration required to achieve a specified temperature rise was reported [71]. A temperature increase alone is not equivalent to photothermal conversion efficiency, which requires a defined heat-balance analysis and appropriate solvent and heat-loss corrections [73]. The available results therefore support photothermal responsiveness and short-term cycling stability but not a quantified mass-efficiency advantage.

#### 5.1.3. Limits of the Degradation Evidence

Du et al. reported an 82.5 ± 4.1% loss of absorbance at 808 nm for PPy@PyCOOH after 60 min in 10 mM H_2_O_2_ at pH 6.5, compared with 17.8 ± 2.6% for pristine PPy under the same conditions [71]. This comparison supports faster optical attenuation and greater oxidative susceptibility under the tested in vitro condition, but absorbance loss alone does not demonstrate biodegradation, mineralization, or in vivo clearance [141]. Standardized biodegradation assessments employ endpoints such as dissolved organic carbon removal, CO_2_ evolution, or oxygen consumption, while polymer degradation investigations may also examine mass loss, molecular weight changes, and transformation products. In vivo clearance is a separate biological outcome that requires biodistribution and elimination measurements, including analysis of organs and excreta [142]. Accordingly, the reported PPy@PyCOOH response should be described as oxidative optical attenuation or increased oxidative susceptibility rather than confirmed biodegradability.

### 5.2. Metal–Polyphenol Networks: Catalytic Gain and Incremental Process Burden

Metal–phenolic network (MPN) formation through tannic acid–copper (TA–Cu) coordination on a preformed PPy@PyCOOH core represents a post-synthetic functionalization stage [71]. Rapid assembly in aqueous media under ambient conditions is a characteristic advantage of many MPN platforms [99]. However, aqueous and low-temperature processing alone do not demonstrate a low overall environmental burden, because additional ligand and metal inputs, particle recovery, washing, material losses, and the subsequent fate of the metal component must also be considered [143,144].

#### 5.2.1. Catalytic Benefit and Additional Processing

In the representative PPy@PyCOOH system, TA–Cu coating produced a 1.52 ± 0.08-fold ROS-probe signal relative to the uncoated core, supporting enhanced H_2_O_2_ activation after interfacial functionalization [71]. This result should not, however, be described as catalytic amplification per added copper atom because the copper content of the final material was not quantified. Moreover, Cu/H_2_O_2_ chemistry can involve Cu(I)/Cu(II) cycling and multiple reactive intermediates; an increased probe signal therefore indicates enhanced oxidizing activity but does not, by itself, identify a single ROS-generating pathway [118].

The coating stage includes coordination, particle recovery, and washing operations after core synthesis. The isolated yield of the coated particles, material loss during recovery, washing-water consumption, and cumulative yield were not reported. The functional enhancement is therefore experimentally supported, whereas the magnitude of the associated process burden remains unquantified.

#### 5.2.2. Copper Utilization and Release: Key Unreported Parameters

Morphological and spectroscopic characterization supported the formation of the TA–Cu interface, but mass-based copper loading, retention after washing, and copper recovery in the supernatant and washing fractions were not reported. Confirmation of copper incorporation is not equivalent to measuring metal utilization efficiency. Such an assessment requires a copper mass balance covering the initial input, isolated particles, reaction supernatant, and wash streams.

MPN composition and stability can vary with metal identity, ligand-to-metal ratio, pH, and assembly conditions [145]. Separate work on TA–Cu-coated urinary catheters has demonstrated that ICP-OES can quantify copper release and that this depends on environmental pH [72]. However, numerical release values from that substrate cannot be transferred to TA–Cu-coated PPy particles. Copper release from the PPy-based formulation was not evaluated in water, PBS, serum-containing medium, or lysosome-mimicking conditions, and dissolved copper was not distinguished from ligand-complexed or particle-associated copper [71]. Future studies should therefore report total copper loading, retention after washing, time-dependent release in biologically relevant media, and the operationally or chemically defined form of released copper.5.2.3 Morphological and Photothermal Trade-Offs.

The uncoated PPy@PyCOOH particles had a reported mean hydrodynamic diameter of 248 ± 12 nm, whereas the highest-input TA–Cu coating formulation reached 462 ± 28 nm and exhibited greater aggregation [71]. This finding supports a formulation-dependent trade-off between interfacial functionalization and colloidal size, although coating thickness was not measured directly. The previously stated 20 nm threshold should therefore be removed.

Both uncoated and coated formulations retained photothermal heating over three 808 nm irradiation cycles, but photothermal conversion efficiency was not calculated [71]. The evidence consequently supports short-term photothermal responsiveness after coating, but not a numerical relationship between coating thickness, reaction time, ROS generation, and PCE. Because nanoparticle aggregation and apparent size may change substantially in protein-containing biological media, colloidal stability should also be evaluated under the actual exposure conditions used in biological experiments (Moore et al., 2015) [146].

### 5.3. Process-Level Comparison: Evidence Boundaries and Reporting Requirements

A quantitative process-level comparison between in situ Py-3-COOH copolymerization and post-synthetic TA–Cu functionalization is not yet available in the literature [71]. For the Fe^2+^/H_2_O_2_-mediated PPy@PyCOOH route, reaction inputs and conditions were reported. However, monomer conversion, comonomer incorporation, isolated dry yield, washing-water consumption, process mass intensity (PMI), E-factor, and residual iron were not quantified. For the subsequent TA–Cu functionalization stage, formation of the metal–polyphenol interface and the associated functional response were demonstrated, whereas coated-particle yield, copper loading, copper retention after washing, material losses during recovery, and copper recovery from process streams were not reported. Numerical comparisons of cumulative yield, waste generation, or metal-utilization efficiency would therefore require unsupported inference [16,59,143,147].

The available evidence, nevertheless, supports a qualitative distinction between processes. In situ copolymerization combines PPy formation and carboxyl functionalization in a single polymerization stage, thereby avoiding a separate post-synthetic carboxylation step. By contrast, TA–Cu assembly introduces an additional functionalization stage after core formation, together with further material inputs, particle recovery, and washing. However, step count alone does not establish a higher E-factor or environmental burden, because these outcomes also depend on solids concentration, recovery efficiency, water and solvent consumption, energy demand, and waste treatment [16,147].

Accordingly, unavailable process parameters should be designated as not reported (NR), rather than replaced with estimates or values transferred from non-PPy systems [59,62]. Future comparisons should use a consistent system boundary and report monomer conversion, isolated yields after each processing stage, total material and water inputs, energy requirements, residual and released metals, and recovery from process streams [16,59,143]. Functional endpoints—including photothermal response, ROS-probe signals, colloidal stability, and oxidative transformation—should remain separately visible rather than being aggregated into a single greenness score [59,62,147]. The minimum reporting checklist proposed in Section 2.4 provides a practical basis for such comparisons [59].

### 5.4. Design Implications: From Functional Addition to Evidence-Based Process Design

First, functional performance should be reported together with the conditions and denominator used for comparison. For photothermal studies, wavelength, irradiance, exposure time, sample volume and geometry, particle concentration, optical absorption, and the heat-balance method should accompany any reported conversion efficiency. Photothermal conversion efficiency alone may also be insufficient for cross-material ranking unless differences in absorption capacity are taken into account [74]. For multifunctional materials, photothermal or catalytic output should additionally be related to the mass of final material and, for metal-mediated activity, to the measured metal content rather than merely to the nominal metal input [148].

Second, a material balance should be closed across both core synthesis and coating. At a minimum, studies should report reagent inputs, monomer conversion where measurable, isolated yield after each processing stage, water and solvent consumption, and the mass of the final product. Metal-containing formulations should additionally report metal loading, retention after washing, metal recovered in process streams, and release under relevant exposure conditions. These data would permit the calculation of E-factor, process mass intensity, and metal utilization efficiency rather than relying on step-count-based estimates [143].

Third, measured values, estimates, and unreported parameters should remain clearly separated. Missing values should be designated as NR rather than replaced with numbers from compositionally or procedurally different systems. Minimum-information initiatives in bio–nano research similarly emphasize complete material characterization and experimental reporting as prerequisites for reproducibility and quantitative comparison [59].

A multicriteria dashboard may eventually integrate process efficiency, functional performance, colloidal stability, metal release, and biological safety. However, a single weighted sustainability score should not be introduced until the criteria, system boundaries, normalization procedures, weights, data quality, and uncertainty are defined transparently. Multicriteria analyses can conceal important trade-offs when aggregation and weighting choices are not disclosed; individual indicators should therefore remain visible alongside any composite result [149].

Functionalization should ultimately be treated as a process-aware design decision. Each additional component should be justified not only by the function it introduces, but also by measured evidence concerning material utilization, processing requirements, stability, and biological and environmental fate.

## 6. Translational and Regulatory Considerations for Sustainable PPy Nanomedicines

### 6.1. Boundaries Between Green Synthesis, Biocompatibility, and Translatability

Green synthesis, biocompatibility, and clinical translatability represent related but non-interchangeable assessment domains. Green synthesis concerns material and energy inputs, reagent hazards, purification, waste generation, and lifecycle boundaries. Biocompatibility describes biological responses to a defined formulation, dose, route, and exposure duration [6,150]. Clinical translatability additionally requires reproducible manufacturing, validated analytical control, pharmacokinetic and biodistribution evidence, long-term safety, and an acceptable regulatory benefit–risk profile.

Consequently, aqueous synthesis does not establish biocompatibility, short-term cell viability does not establish systemic safety, and therapeutic efficacy in a mouse xenograft does not establish translational readiness [151,152]. These domains should be reported separately before their relationships are interpreted.

### 6.2. Limitations of Current Green Metrics in Nanomedicine

Mass-based metrics such as atom economy, PMI, and E-factor are essential for evaluating process efficiency. However, they do not independently capture nanoparticle aggregation, biological identity, tissue exposure, immune interaction, or long-term persistence [19]. Conversely, favorable cytocompatibility or therapeutic performance cannot compensate for an unreported process inventory [153].

Water should not automatically be treated as burden-free because washing, centrifugation, purification, and wastewater treatment may dominate the process [143]. Likewise, performance-normalized metrics can supplement but should not replace mass-based results, because the selected biological endpoint and assay conditions may substantially affect the apparent efficiency. The dual-axis approach should therefore retain process-related sustainability and biofunctional evidence as separate outputs rather than combining them into a weighted overall score [59].

### 6.3. Manufacturing Reproducibility, Scale-Up, and GMP Readiness

Translation requires defining critical material attributes and critical process parameters. For PPy nanomedicines, relevant material attributes include particle-size distribution, aggregation state, morphology, surface chemistry, doping state, residual pyrrole, oxidant and metal content, endotoxin and sterility status, photothermal response, ROS-related activity, and metal or payload release [154]. Process parameters include reagent-addition rate and order, mixing, pH, temperature, reaction time, purification, drying, and sterilization.

Scale-up may alter mixing, heat transfer, nucleation, and local reagent concentration, leading to batches that differ from laboratory preparations even when nominal reagent ratios remain unchanged. Development should therefore include independent-batch reproducibility, validated orthogonal analytical methods, storage stability, sterilization compatibility, and predefined acceptance ranges [155,156]. Current regulatory guidance treats nanomaterial-containing drug products through a product-specific, risk-based approach. It places particular emphasis on physicochemical characterization, process control, stability, and links between material attributes and clinical performance [157].

### 6.4. Pharmacokinetics, Biodistribution, Biotransformation, and Clearance

Pharmacokinetic evidence remains limited and highly formulation-specific for PPy nanomedicines [158]. Ultrasmall PPy–PEG nanoparticles of approximately 2 nm have been reported to undergo renal clearance [35]. However, this finding should not be generalized to larger PPy particles, hollow nanospheres, aggregates, or metal-containing formulations [159]. Size, protein adsorption, surface charge, colloidal stability, and degradation can alter circulation and organ distribution [142,160,161].

A further analytical limitation is that fluorescence, drug, or metal labels may dissociate from the carrier, thereby failing to reflect the fate of the PPy backbone accurately [162,163,164]. Future studies should separately track the polymer-associated signal, therapeutic payload, Fe or Cu content, and soluble degradation products using orthogonal analytical methods [163,164,165]. Blood pharmacokinetics should be combined with time-resolved measurements in the tumor, liver, spleen, kidney, lung, urine, and feces [158,166].

Optical attenuation, particle fragmentation, and loss of TEM-visible morphology should be described as physicochemical transformation unless polymer-chain scission and product formation are directly demonstrated [165,167]. Complete clearance requires recovery or accounting for the material and its transformation products, rather than the mere disappearance of an optical signal [162,164,165,166].

### 6.5. Toxicology, Immune Interaction, Long-Term Fate, and Regulatory Integration

Existing studies provide useful but formulation-limited safety evidence. A six-week mouse study reported no detectable inflammatory or immune-related effects for the specific chemically prepared PPy particles investigated [34]. By contrast, size-controlled PPy nanoparticles produced size- and dose-dependent effects on viability, oxidative stress, and macrophage responses in vitro [88]. Together, these studies demonstrate that biological response depends on particle properties and exposure conditions and that “PPy biocompatibility” cannot be treated as a material-wide constant.

Future safety studies should evaluate hemolysis, coagulation, complement activation, cytokine release, oxidative stress, macrophage uptake and activation, genotoxicity, and histopathology after both single and repeated administration [168,169,170,171]. For Fe- or Cu-containing systems, toxicity associated with the polymeric particles should be distinguished from that associated with released metal species and degradation products [169,170]. Follow-up periods should extend beyond short-term tumor regression to include recovery, organ burden, chronic inflammation, and delayed toxicity [169,172].

**Figure 4:**
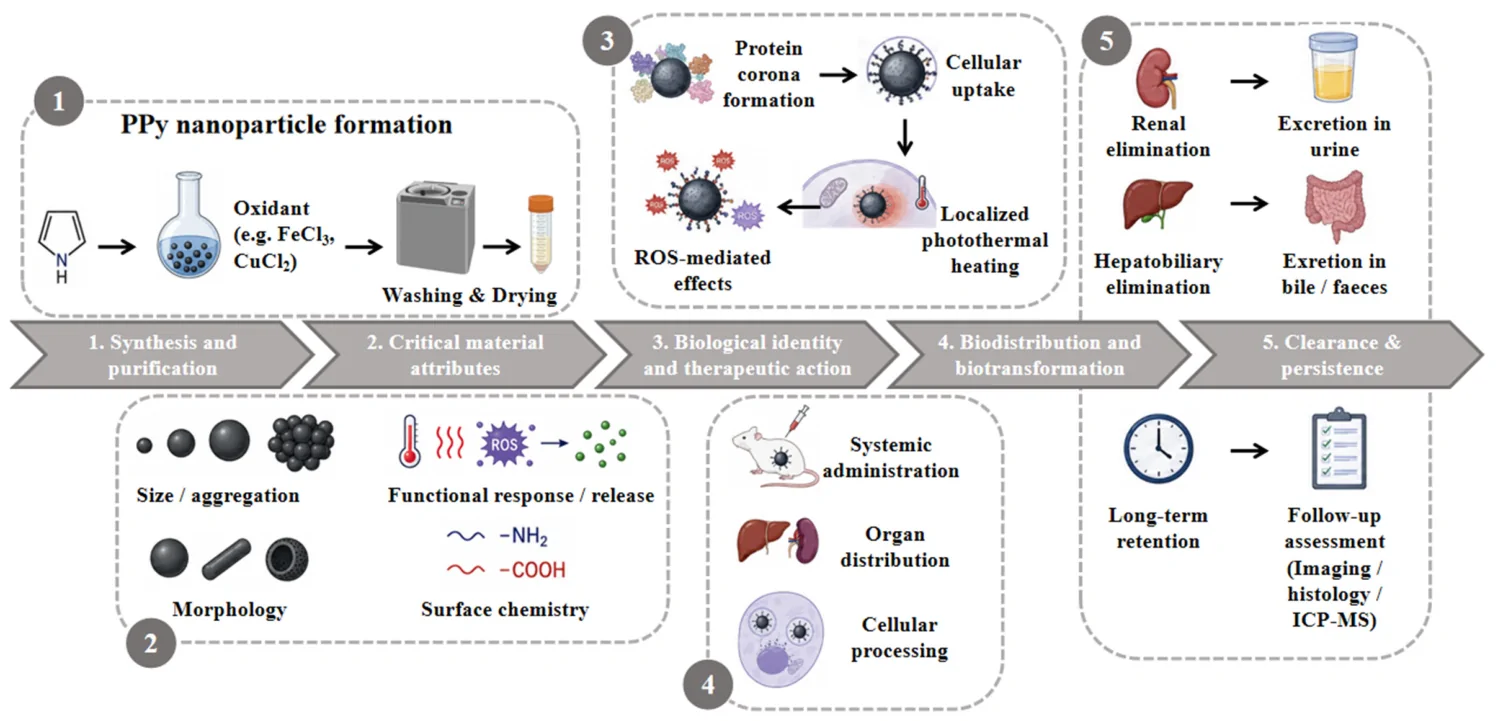
Evidence pathway for the translational assessment of PPy nanomedicines, linking synthesis and functionalization to critical material attributes, biological action, biodistribution, biotransformation, clearance, and long-term fate. The PPy backbone, therapeutic payload, metal components, and degradation products should be tracked separately. Loss of optical signal or particle morphology does not establish biological clearance.

PPy nanomedicines must ultimately be assessed as complete therapeutic systems, including the nanoparticle formulation, any loaded drug or metal component, and the irradiation device and treatment protocol (Figure 4) [15,173]. Wavelength, power density, exposure duration, beam geometry, and thermal dose therefore form part of the translational evidence package. Current European regulatory analysis similarly emphasizes particle characterization, scale-up, impurities, nano-specific biodistribution, immune responses, altered pharmacokinetics, and product-specific regulatory assessment.

Future studies should apply the minimum reporting protocol proposed in Section 2.4 to quantify batch inventory, mass and energy intensity, metal balance, functional performance, degradation and lifecycle boundaries. This approach would enable auditable inter-study comparison without collapsing green-synthesis and biofunctional outcomes into a subjective weighted score [59,65].

## 7. Conclusions

This review set out to re-evaluate polypyrrole (PPy)-based nanomedicines not merely as photothermally active platforms, but as model systems for integrating green chemistry principles into cancer theranostics. The central argument developed across the preceding chapters is that sustainability and therapeutic performance are not opposing objectives—rather, they can be coupled through route-driven, structure-informed design. A key premise of this work is that oxidant selection is an important process variable that can influence PPy formation, residual metal content, morphology, and subsequent functionalization requirements. FeCl_3_-mediated routes provide a comparatively direct pathway to photothermally active PPy but generally require additional components when substantial catalytic ROS generation or other redox functions are desired. CuCl_2_-based systems embed redox-active copper centers, enabling catalytic ROS amplification and chemodynamic synergy, yet require careful quantification of copper incorporation, speciation, retention, release, and long-term biological disposition. Fe^2+^/H_2_O_2_-mediated copolymerization represents a functionally distinct strategy that has been used to introduce carboxyl functionality, hollow morphology, and oxidation-responsive optical behavior during PPy core formation, while potentially reducing reliance on deliberately retained redox-active metal components.

The comparison does not support a universal sustainability ranking of FeCl_3_-, CuCl_2_-, and Fe^2+^/H_2_O_2_-mediated routes. Instead, their relative suitability depends on the intended therapeutic function, residual-metal burden, functionalization requirements, and the amount of additional processing needed to obtain the final nanomedicine. Post-synthetic coupling introduces additional reagents, separation and washing operations, whereas in situ copolymerization can integrate reactive functionality during particle formation. Fe^2+^/H_2_O_2_-mediated copolymerization with Py-3-COOH has demonstrated direct incorporation of carboxyl functionality and, in the reported formulation, was associated with hollow-particle formation and enhanced oxidative susceptibility. TA–Cu metal–polyphenol interfaces provide additional catalytic functionality, but their sustainability assessment requires quantitative data on coating yield, copper loading, retention during washing, release in biological media, and the influence of coating thickness on photothermal performance. The analysis further identifies systemic reporting deficiencies: process-level indicators such as isolated yield, PMI, E-factor, washing demand, metal-utilization efficiency, and complete mass balances remain infrequently reported in the PPy nanomedicine literature surveyed. Sustainability claims remain difficult to compare without process-level mass and energy data, whereas degradability claims require direct characterization of structural change, soluble products, residual particles, and mass recovery.

To improve comparability, this review introduces two complementary tools: a dual-axis evidence framework that separates process sustainability from therapeutic functionality, and a tiered PPy-specific reporting checklist that identifies the minimum data needed for progressively more quantitative assessment. This framework enables transparent comparison and reveals where data gaps persist. Based on this integrated analysis, five actionable design principles emerge: (1) prefer aqueous, room-temperature oxidative polymerization; (2) minimize intentionally retained redox-active metals unless their therapeutic contribution is quantitatively demonstrated and their release and fate are adequately characterized; (3) prioritize degradability mechanisms intrinsic to the PPy-containing structure, while separately evaluating the fate of detachable coatings and degradation products; (4) employ natural polyphenols for metal coordination with strict attention to metal retention and leaching; and (5) normalize therapeutic performance to relevant material, metal and energy inputs, while reporting the complete experimental conditions used for comparison.

Future work should combine standardized degradation validation with molecular-weight analysis, transformation-product identification, carbon and metal mass balances, and testing under physiologically relevant conditions. Metal-containing formulations require routine measurement of metal retention, speciation, release kinetics, and relationships between exposure and toxicity. Process-level reporting should include conversion, isolated and cumulative yield, washing demand, solvent recovery, PMI, E-factor, and measured or transparently estimated energy consumption. These assessments should be accompanied by independent-batch reproducibility, scalable purification, pharmacokinetics, biodistribution, repeated-dose safety, immune interaction, and evaluation of the complete nanoparticle–payload–irradiation system.

PPy-based nanomedicines therefore offer a meaningful opportunity to align cancer theranostics with green chemistry principles, provided that synthesis and functionalization are treated as coupled design dimensions. Process sustainability, biological safety, and translational readiness should remain distinct but interconnected evaluation domains. Future PPy systems should be judged through multi-objective performance rather than optimization of a single photothermal, catalytic, or biological endpoint. Sustainability-guided design should be regarded not as a constraint on innovation, but as a means of improving the precision, safety, manufacturability, and translational credibility of PPy-based nanomedicines.

## Figures and Tables

**Table 2:** Tiered quantitative evaluation protocol for PPy-based nanomaterials.

Tier and Domain	Minimum Information to Report	Quantitative Output or Interpretation Rule	References
I—Process boundary and inventory	Batch scale; starting and ending boundaries; mass of monomer, oxidant, dopant, metal salt, template, stabilizer, solvent, water and purification media; recovered inputs	Complete batch inventory and declared reference flow; washing and dialysis media must not be omitted	[16,63,64,65]
I—Product, conversion and yield	Dry product mass; drying conditions; moisture, dopant and metal content; residual monomer; independent batch number	Report monomer conversion separately from isolated yield; do not infer conversion from recovered mass when dopants, counterions or metals contribute to product mass	[46,63,69,70]
II—Mass and solvent metrics	Complete input and waste inventory; recovered solvent or reagent; dry application-ready product mass	PMI, E-factor, water intensity and organic-solvent intensity; distinguish water-inclusive and water-excluded values	[21,44,63,64,66]
II—Atom economy	Balanced reaction equation and defined composition of the desired doped PPy product	Calculate on a declared repeat-unit basis only when stoichiometry is defined; otherwise report not calculable	[21,63,67]
II—Energy demand	Temperature, duration, equipment power and scale for reaction, stirring, sonication, centrifugation, hydrothermal processing and drying	Energy intensity in kWh g^−1^ dry product; identify measured versus rated-power-derived values	[44,65]
II—Metal utilization and release	Metal charged; final metal content; metal in supernatant, washing fractions and solid residues; digestion and analytical method; release medium, pH, temperature and duration	Metal retention, metal-balance closure and cumulative release relative to experimentally measured initial loading; feed ratio alone is insufficient	[59,68,71,72]
II—Photothermal and ROS performance	Wavelength, incident power, concentration, volume, absorbance or extinction, optical geometry, background correction and heating–cooling curves; ROS species, probe, calibration, controls and replicates	PCE or external conversion efficiency with full calorimetric context; species-specific ROS output rather than unqualified “total ROS”	[73,74,75,76]
III—Degradation and biological safety	Mass loss, molecular-weight change, degradation products, TOC, metal release and exposure medium; material characterization in biological media, dose, time, controls and replicates	Degradation kinetics, carbon/metal balance and dose-dependent safety outcomes; TEM disruption alone does not establish complete degradation or clearance	[59,68,76,77]
III—Lifecycle and evidence status	Upstream reagents, electricity mix, water and solvent supply, drying, waste treatment, allocation assumptions, uncertainty and raw-data availability	Cradle-to-gate profile following ISO principles; label evidence as directly reported, transparently calculated, external benchmark, NR or not calculable	[59,78]

**Table 3:** Evidence-separated comparison of process-related and functional metrics for PPy@PyCOOH and TA–Cu-coated PPy@PyCOOH, with quantitative benchmarks from related PPy and TA–Cu systems.

Metric	PPy@PyCOOH: Direct Evidence	TA–Cu-Coated PPy@PyCOOH: Direct Evidence	Cross-Study Quantitative Benchmark and Interpretation
**Synthesis route and reported inputs**	Py-3-COOH (33.3 mg) and NaOH (1.2 mg) were dissolved in 5 mL water, followed by 0.5 mL FeCl_2_·4H_2_O solution (26 mg mL^−1^), 83 μL pyrrole and 300 μL of 30 wt% H_2_O_2_. Polymerization proceeded for 3 h at room temperature [71]	A 25.5 mg mL^−1^ TA solution was mixed with formaldehyde at a 2:1 volume ratio. Different amounts of this solution were added to ethanol/water/ammonia media and reacted with PPy@PyCOOH for 24 h. Cu(NO_3_)_2_·3H_2_O was subsequently added at a nominal Cu:PPy@PyCOOH mass ratio of 1:1, followed by hydrothermal treatment at 100 °C for 24 h [71]	These are directly reported batch inputs. The initial Cu feed ratio must not be interpreted as final Cu loading or retention. Metal–polyphenol network formation is strongly dependent on pH, metal-to-ligand ratio and assembly conditions [99]
**Monomer conversion**	NR	Not applicable to the coating reaction; conversion of the PPy@PyCOOH precursor was NR	In aqueous FeCl_3_-mediated PPy synthesis, pyrrole conversion varied from 31.18% to 89.83% depending on FeCl_3_ concentration and temperature [46]. Kinetic studies further demonstrate strong dependence on temperature and oxidant concentration [70]. H_2_O_2_-mediated PPy formation has also been demonstrated, but under conditions different from the Fe^2+^/H_2_O_2_-mediated Py/Py-3-COOH copolymerization used here [132]
**Isolated or cumulative yield**	NR	NR	After purification, yields of 74%, 56% and 63% were reported for solvent-free FeCl_3_-mediated synthesis of PPy, poly(N-methylpyrrole) and poly(N-ethylpyrrole), respectively [69]. These values demonstrate monomer- and process-dependent yield variation and are not estimates for PPy@PyCOOH.
**Purification and post-processing burden**	Repeated centrifugation and washing were reported, but the number of cycles, total washing-water volume and recovered dry mass were not specified for PPy@PyCOOH [71]	The coating introduced ethanol, water, ammonia, formaldehyde, an additional 24 h reaction and 24 h treatment at 100 °C. Final washing inputs and recovered product mass were not quantified [71]	The available evidence permits a qualitative comparison of processing complexity but not a quantitative comparison of material efficiency.
**Process mass intensity and E-factor**	NR and not calculable because the final isolated product mass, total washing inputs and waste outputs were not reported [71].	NR and not calculable for the same reasons, with additional uncertainty from coating and hydrothermal-processing inputs [71].	PMI requires the total mass of all process inputs per mass of isolated product, whereas the E-factor requires the mass of waste per mass of product [16,21,63,64]. Industry-average values or values from unrelated nanomaterials should not be transferred to these formulations.
**Metal incorporation and retention**	FeCl_2_·4H_2_O was used during synthesis, but residual Fe content in the recovered PPy@PyCOOH particles was NR [71].	A nominal Cu:PPy@PyCOOH feed ratio of 1:1 was reported, but final Cu loading, Cu retention after processing and Cu content in the supernatants were NR.	ICP-OES measurements gave 3.10 ± 0.10 wt% Cu for an isolated molecular TA–Cu complex [133], whereas CuTA nanosheets contained 30.9 wt% Cu [134]. This wide difference illustrates the dependence of Cu loading on stoichiometry and material architecture; neither value represents the present coating.
**Cu release**	Not applicable.	NR	In a different TA–Cu coating system, single- and double-layer coatings contained 0.675 ± 0.075 and 2.333 ± 0.063 μg Cu cm^−2^, respectively. The double-layer coating released 0.499, 1.105 and 0.907 μg Cu cm^−2^ over 7 days at pH 7.4, 4.0 and 10.0, respectively [72]. These results demonstrate that Cu release must be measured for each formulation and environment.
**Photothermal heating response**	The dispersion reached approximately 34 °C within 5 min under 808 nm irradiation at 1 W cm^−2^ and 100 μg mL^−1^. Nearly overlapping curves were obtained over three heating–cooling cycles.	Under identical conditions, the maximum temperature plateaued at approximately 30–31 °C, with reproducible heating over three cycles.	The directly comparable data indicate an approximately 3–4 °C reduction in maximum temperature after coating. Final temperature, however, is not equivalent to numerical photothermal conversion efficiency.
**Numerical photothermal conversion efficiency**	NR; the heat-transfer coefficient, solvent-background contribution and other energy-balance parameters required for calculation were not provided [75].	NR for the same reason.	At 808 nm, reported PCE values include 44.7% for sub−100 nm PPy nanoparticles [24], 55.6% for ultrathin PPy nanosheets [29] and 33.35% for approximately 2 nm PPy–PEG nanoparticles [35]. These values constitute formulation-specific benchmarks rather than an estimated PCE for the target systems.
**ROS-related response**	PPy@PyCOOH showed a pH-dependent fluorescence response, although the conventional TMB assay was affected by precipitation and could not provide a reliable colorimetric comparison.	TA–Cu functionalization was reported to increase ROS yield to 1.52 ± 0.08-fold relative to the study control and produced consistently stronger fluorescence responses across the investigated pH range.	Cu–TA interfaces have independently been shown to catalyze H_2_O_2_ decomposition and hydroxyl-radical formation, with photothermal heating further enhancing ROS production [135]. This supports the proposed mechanism but does not replace formulation-specific measurement.

## Data Availability

No new data were created or analyzed in this study. Data sharing is not applicable.
